# Design of a water-soluble chitosan-based polymer with antioxidant and chelating properties for labile iron extraction

**DOI:** 10.1038/s41598-023-34251-3

**Published:** 2023-05-16

**Authors:** Coralie Grange, Axel Aigle, Victor Ehrlich, Juan Felipe Salazar Ariza, Thomas Brichart, Fernande Da Cruz-Boisson, Laurent David, François Lux, Olivier Tillement

**Affiliations:** 1 MexBrain, 13 avenue Albert Einstein, Villeurbanne, France; 2 Institut Lumière-Matière, UMR 5306, Université Lyon1-CNRS, Université de Lyon, Villeurbanne Cedex, France; 3 Ingénierie des Matériaux Polymères, CNRS UMR 5223, Univ Claude Bernard Lyon 1, Institut national des Sciences Appliquées, Université Jean Monnet, Univ Lyon, 15 bd Latarjet, 69622 Villeurbanne, France; 4 Institut Universitaire de France (IUF), 75231 Paris, France

**Keywords:** Drug discovery, Chemistry

## Abstract

Loosely bound iron, due to its contribution to oxidative stress and inflammation, has become an important therapeutic target for many diseases. A water-soluble chitosan-based polymer exhibiting both antioxidant and chelating properties due to the dual functionalization with DOTAGA and DFO has been developed to extract this iron therefore preventing its catalytic production of reactive oxygen species. This functionalized chitosan was shown to have stronger antioxidant properties compared to conventional chitosan, improved iron chelating properties compared to the clinical therapy, deferiprone, and provided promising results for its application and improved metal extraction within a conventional 4 h hemodialysis session with bovine plasma.

## Introduction

Iron is a trace element involved in numerous enzymatic reactions within metabolism as well as oxygen transport^1^. However, due to its ability to undergo redox reactions and produce reactive oxygen species (ROS) through the Fenton reaction, an excess of iron can cause oxidative damage to DNA, lipids, and/or proteins and is toxic for cells and organs^2^.

$$Fe^{2 + } + H_{2} O_{2} \to Fe^{3 + } + HO^{ \bullet } + OH^{ - } \left( {Fenton \;\;reaction} \right)$$

Interestingly, in humans, excess iron is not naturally excreted via the fecal or renal route like other essential dietary metals. The homeostasis of iron is instead controlled entirely by proteins through the processes of adsorption, circulation, storage, and recycling^3^. Considering the essential and toxic nature of iron, a dysregulation of iron metabolism can have a harmful impact on health and lead to the onset of various diseases.

Iron-overload diseases are frequently genetic in nature. For example, in the case of primary hemochromatosis, mutations in the genes encoding hepcidin lead to an inability to regulate dietary iron absorption and results in the accumulation of iron in various organs like the liver or the heart^4,5^. Since most of the proteins involved in iron metabolism are produced in the liver, like hepcidin and transferrin, chronic liver diseases are also associated with iron overload. Increased iron levels were observed in patients with alcoholic liver diseases, nonalcoholic fatty liver disease, and hepatitis C viral infection^6^. In addition, it has been observed that patients with hereditary hemochromatosis are at higher risk to develop liver cancer compared to patients with non-iron-related chronic liver disease^7^. Several studies have associated the dysregulation of iron homeostasis with mutagenesis and enhanced tumor growth^8,9^. Those suffering from iron overload show elevated risk of bacterial infections due to the important role that iron plays in pathogenic development, and several mechanisms of incorporation have been identified in bacterial species including the secretion of siderophores to bind excess ferric iron^10^. Iron overload has also been suspected to be implicated in pathologies where oxidative stress plays an important role. An example of this is the correlation of iron and neurodegenerative diseases such as Alzheimer’s disease and Parkinson’s disease, where excessive labile iron within the brain can lead to protein aggregation and increase the production of reactive oxygen species which further deteriorates the dopaminergic neurons^11^.

Excess iron in localized regions has also been associated with some pathologies like ischemic stroke^12^. An increase of labile iron can also occur in the case of acute injuries, as observed in intensive care units (ICU). Acute kidney injury (AKI) is a severe and frequent complication in hospitalized patients (18%) with a high mortality which can progress into end-stage renal disease (ESRD)^13^. Various procedures can release catalytic iron, like cardiopulmonary by-pass and ischemia and reperfusion which contributes to oxidative damage and kidney injury^14,15^. A dysregulation of iron homeostasis has also been observed in patients suffering from acute-on-chronic liver failure (ACLF), which often leads to multi-organ failure and mortality in 50–90% of cases^16^.

These examples highlight the importance of a well-regulated iron homeostasis and the benefit of extracting any excess of endogenous loosely bound iron found within the plasma or in localized regions. Phlebotomy, and more commonly, chelation therapy are the principal treatments for iron overload diseases. Chelation therapy targets toxic metal ions to neutralize and promote their excretion^1^. Deferoxamine (DFO), a natural siderophore, was the first drug used for treating secondary hemochromatosis, however, current administration of DFO by subcutaneous infusion (8–12 h for 5–7 times a week) is associated with frequent adverse effects leading to low patient compliance^17,18^. An alternative to DFO is deferiprone, an orally active chelating agent that can form a 3:1 complex with iron (III)^19^. Due to its moderate plasma half-life (less than 2 h), the common administration of deferiprone is 75 mg.kg^−1^ per day, divided into 3 doses^20^. Despite the high efficiency for iron scavenging, current chelating-based therapies are limited in the case of localized iron overload due to the adverse effects associated with their systemic action and the required therapeutic dose. Thus, iron chelation remains a challenge, in particular in intensive care units (ICU) where the release of redox active iron is a critical parameter that requires rapid treatment. Leaf et al.^21^ demonstrated that an elevated plasma catalytic iron level in patients on arrival to the ICU is correlated with a greater risk of AKI and hospital mortality. In these situations, renal replacement therapy (including continuous venovenous hemodialysis) has become a standard of care for critically ill patients suffering from AKI^22^.

**Figure 1:**
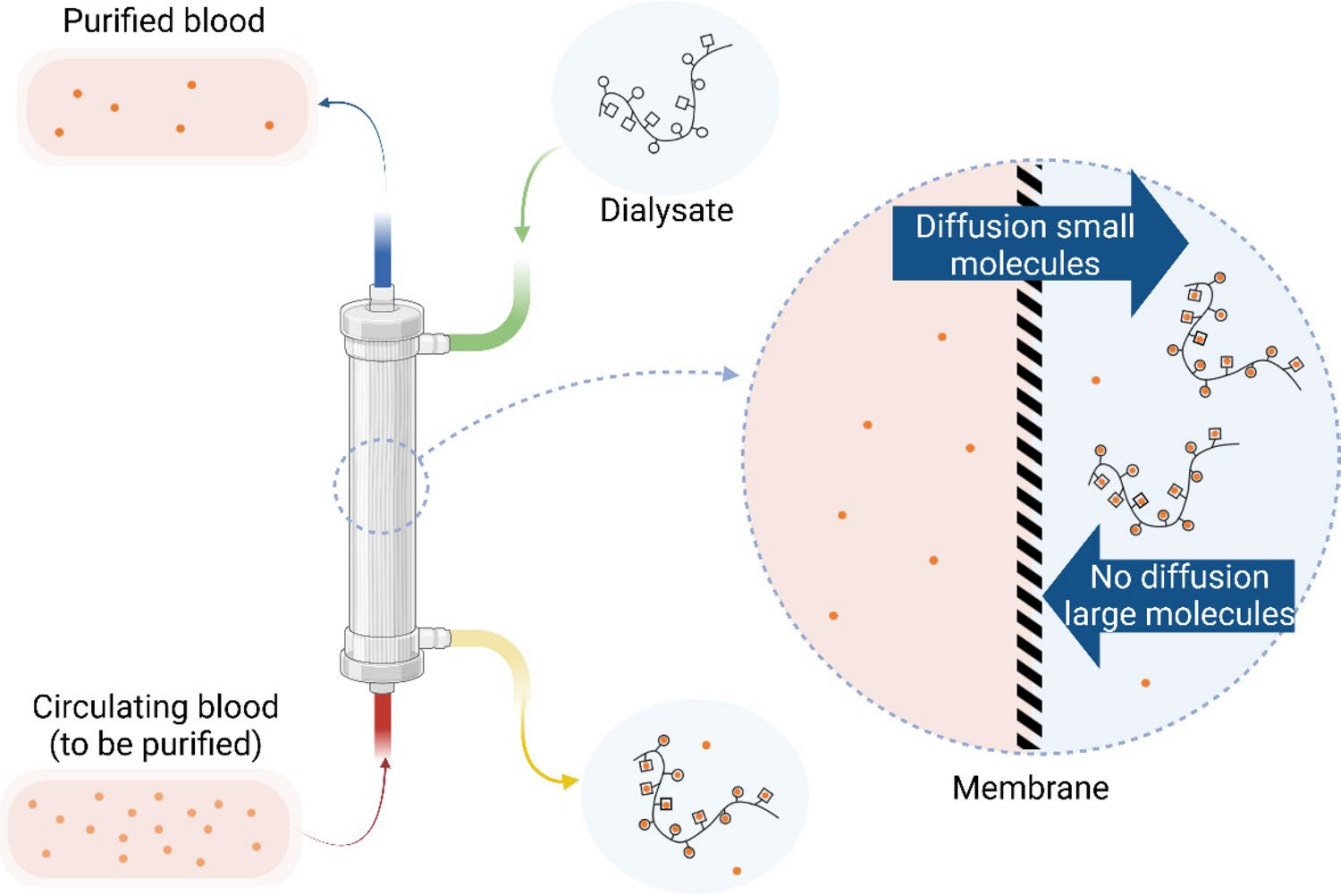
Schematic representation of hemodialysis associated with a double-grafted chelating polymer for metallic extraction of the blood (created with BioRender.com). During hemodialysis, chelating polymers cannot pass through the dialysis membrane in contrary to metallic ions loosely bound to small molecules. Metallic ions are then trapped by chelating polymers and are concentrated in dialysate.

This article aims to present the development of a versatile polymer (Chito@DOTAGA@DFO) with antioxidant activity and chelating properties to extract loosely bound iron therefore preventing the production of reactive oxygen species. The first application of this polymer is its introduction within the dialysate of a hemodialysis cartridge to improve metal extraction during conventional dialysis sessions (Fig. 1). The polymer is advantageous since it (1) remains within the dialysate without passing to the circulating blood and (2) chelates the loosely bound iron that can easily pass through the dialysis membrane. The polymer proposed for this application is a re-acetylated chitosan functionalized with DOTAGA (1,4,7,10-tetraazacyclododececane,1(glutaric acid)-4,7,10-triacetic acid) and DFO (*N*-[5-[[4-[5-[acetyl(hydroxy)amino]pentylamino]-4-oxobutanoyl]-hydroxyamino]pentyl]-*N*′-(5-aminopentyl)-*N*′-hydroxybutanediamide). DFO was grafted to the previously described Chito@DOTAGA^23^ polymer to improve its affinity for iron (III) and its antioxidant properties.

This article describes the three-step synthesis of Chito@DOTAGA@DFO. The polymer’s chelation performance is then assessed in competition with commercial deferiprone, the polymer’s antioxidant properties are assessed in vitro, and the polymer’s implementation into a conventional 4 h hemodialysis session of bovine plasma showed its feasibility and improved metal extraction compared to traditional hemodialysis.

## Results

### Functionalization and characterization of chitosan polymer

**Figure 2:**
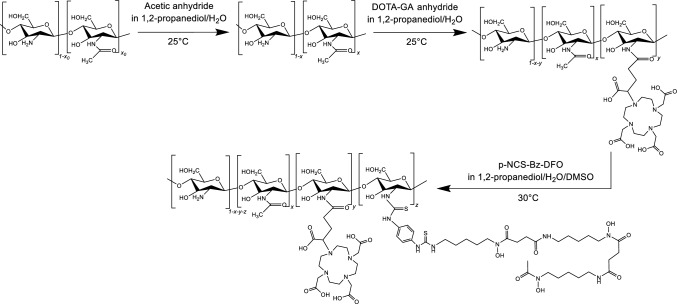
Synthesis pathway of Chito@DOTAGA@DFO from chitosan (x_0_ and x correspond to degrees of acetylation, y to grafting ratio of DOTAGA and z to grafting ratio of p-NCS-Bz-DFO).

**Figure 3:**
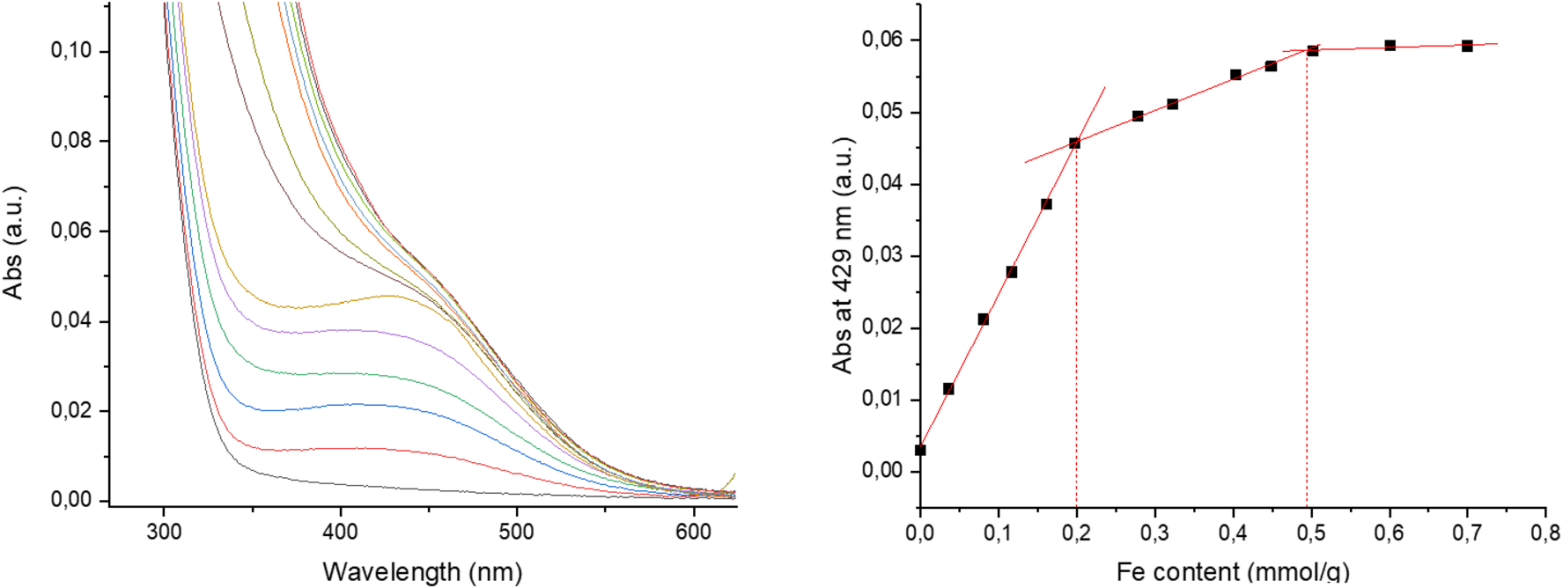
Dosage of a solution of 0.1 g.L^−1^ Chito@DOTAGA@DFO by Fe(III) in acetate buffer (0.1 M ammonium acetate, 0.1 M acetic acid) using UV–Vis spectroscopy and plotting absorbance at 429 nm.

Chito@DOTAGA@DFO was synthetized by addition of functionalized Deferoxamine, p-NCS-Bz-DFO, onto a previously synthetized and characterized Chito@DOTAGA polymer (Fig. 2)^23^. Briefly, Chito@DOTAGA was synthesized in two steps. Raw chitosan (with an initial degree of acetylation x = 0.05 and molecular weight of 2.6 × 10^5^ g mol^−1^) was re-acetylated by dropwise addition of acetic anhydride in a water/1,2-propanediol mixture. An acetylation degree x = 0.29 ± 0.02 was determined by ^1^H NMR by Hirai method^24^ (see Fig. S1 in Supporting Info). The second step was DOTAGA grafting by addition of DOTAGA anhydride onto the previous acetylated chitosan. After purification by tangential filtration (cut-off: 100 kDa), Chito@DOTAGA was freeze-dried and DOTAGA grafting was determined by UV–Vis titration in the presence of increasing concentrations of Cu(II) yielding y = 0.077 (Fig. S2). For the synthesis of Chito@DOTAGA@DFO, the Chito@DOTAGA polymer was first solubilized in water at 10 g L^−1^ and the pH was adjusted to 6 ± 0.5 to ensure that the amine groups of chitosan are deprotonated for the reaction with the NCS function of p-NCS-Bz-DFO. The polymer solution was diluted by a mixture of 1,2-propanediol and DMSO to reduce water fraction and avoid the precipitation of p-NCS-Bz-DFO due to its rather low solubility in water. Similarly, the functionalized DFO (solubilized in DMSO) was added dropwise to avoid its precipitation before successful grafting onto the polymer to maximize the grafting yield. After a second purification by tangential filtration (cut-off membrane of 100 kDa), Chito@DOTAGA@DFO was freeze-dried. The functionalization degree with DFO was assessed by UV–Vis titration of DFO-Fe(III) complex at 429 nm. Increasing concentrations of Fe(III) were added to a Chito@DOTAGA@DFO solution at 0.1 g.L^−1^. For each addition, the absorbance at 429 nm was plotted against the iron concentration (in mmol per gram of polymer) in solution. Provided that DFO shows a larger complexation constant for iron (III) than DOTA (log K_DFO_ = 41.8^25^ and log K_DOTA_ = 29.4^26^), iron will be complexed by DFO first, then DOTAGA. Indeed, on Fig. 3, we observed an increasing absorbance at 429 nm, which is the maximum absorption wavelength of DFO-Fe(III) complex (see Fig. S3). Here, from the first slope, a molar extinction coefficient of 2120 ± 36 L.mol^−1^.cm^−1^ was calculated which is consistent with the molar extinction coefficient found in literature for DFO-Fe(III) complex at 429 nm (ε = 2300 L.mol^−1^.cm^−1^)^27^. The slope discontinuity observed at 0.2 mmol.g^−1^ corresponds to the saturation of DFO sites with Fe(III). This DFO content per gram of polymer corresponds to a DFO grafting rate of z = 0.054 (see details in Supporting Info). A verification titration with Cu(II) was also performed using absorption at 295 nm to determine DOTAGA grafting as the first slope discontinuity. Given that DOTA has a higher complexation constant for Cu(II) than DFO (log K_DFO-Cu(II)_ = 13.5^28^ and log K_DOTA-Cu(II)_ = 22.3^29^), copper will be complexed by DOTAGA first, then DFO. This titration showed a DOTAGA grafting rate y = 0.078 which is consistent with the previous characterization of Chito@DOTAGA before functionalization with DFO (see Fig. S4). DFO functionalization was also confirmed by ^1^H NMR analysis where similar grafting rates were calculated: z = 0.046 or z = 0.044 according to the reference signal chosen for p-NCS-Bz-DFO (aromatic protons at 7.8 ppm or 18 protons from CH_2_ groups between 1.6 and 2.2 ppm, see details in Supporting Info, Figs. S5 and S6).

### Antioxidant power of Chito@DOTAGA@DFO

**Figure 4:**
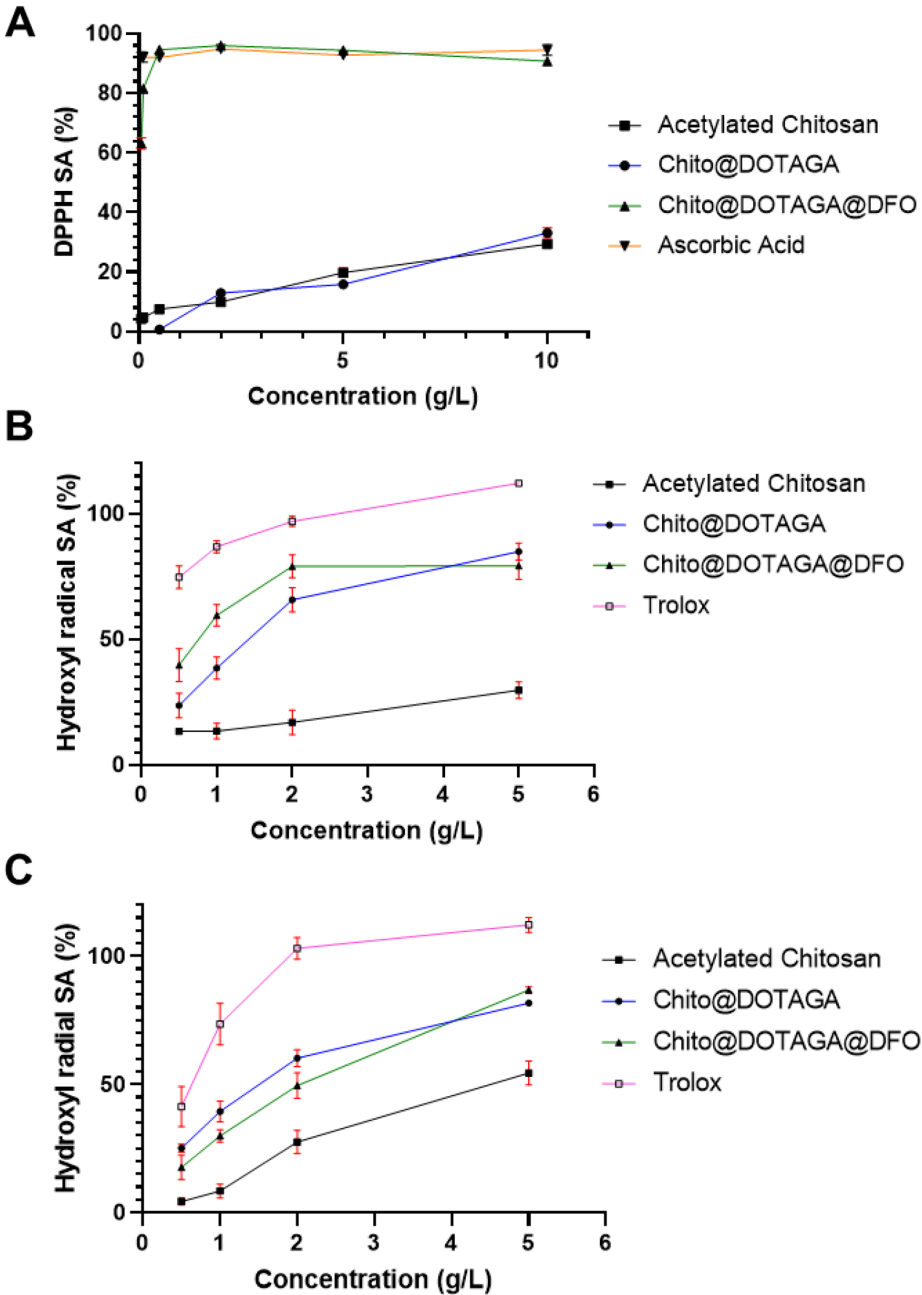
Comparison of antioxidant properties of functionalized chitosan (square: acetylated chitosan DA = 29%; circle: Chito@DOTAGA; triangle: Chito@DOTAGA@DFO) with ascorbic acid (upside-down triangle) and Trolox (empty square) against various radicals. (**A**) Scavenging ability (SA) towards DPPH after 1 h of reaction. (**B**) Hydroxyl scavenging ability by Fenton Generation. (**C**) Hydroxyl scavenging ability by TiO_2_/UV generation.

The antioxidant activity of functionalized chitosan was assessed and compared with reference antioxidants, ascorbic acid or Trolox, a water soluble analogue of vitamin E. DPPH assay is widely used to estimate the activity of antioxidants compounds^30^. DPPH is a stable free radical with a characteristic absorption band around 517 nm, which can be reduced in the presence of a hydrogen donor, leading to a loss of its purple color^31^. Fig. 4A shows the dose effect of chitosan-based polymer on DPPH scavenging ability (SA) after 1 h of reaction. Acetylated chitosan and Chito@DOTAGA show a DPPH scavenging ability of 29% and 33%, respectively, at the highest tested concentration (10 g.L^−1^). Chito@DOTAGA@DFO shows a scavenging ability around 90–95% from 0.5 to 10 g.L^−1^, which is similar to the positive control, ascorbic acid. As a result, functionalization of chitosan with DOTAGA does not have a major impact on DPPH scavenging ability but the addition of DFO significantly improves its DPPH scavenging ability. Figure S7 confirms that the high DPPH scavenging ability observed for Chito@DOTAGA@DFO is mainly due to the presence of DFO on the polymer since chitosan grafted with only DFO (Chito@DFO) and DFO mesylate have similar scavenging abilities as Chito@DOTAGA@DFO. After 19 h of reaction (Fig. S8), improved scavenging abilities are obtained for acetylated chitosan (75% at 10 g.L^−1^) and Chito@DOTAGA (84% at 10 g.L^−1^). However, slightly lower SA are obtained for Chito@DOTAGA@DFO: at 10 g.L^−1^, 91% at one hour vs. 83% after 19 h. One explanation may be the fact that almost all radicals were scavenged after one hour so that the absorbance of the sample is stable between 1 and 19 h whereas the control sample without antioxidants (used for calculation of SA, see formula in “Material and methods”) decreases over time due to DPPH degradation.

Hydroxyl radicals scavenging ability was also tested in two different conditions: hydroxyl generation by Fenton-Reaction or photo-catalysis under UV with TiO_2_. In both cases, hydroxyl scavenging can be measured by the hydroxylation of sodium benzoate which produces hydroxybenzoate that has fluorescence properties (excitation at 310 nm, emission at 407 nm). Scavenging abilities are calculated by comparing the fluorescence intensities between samples with or without antioxidant (negative control). In the first test, hydroxyl radicals are generated in situ by Fenton reaction in the presence of Fe(II) and hydrogen peroxide. This test was performed at 37 °C and pH 7.4 to mimic physiological conditions. Figure 4B shows that all of the functionalized chitosans and Trolox have a dose-dependent hydroxyl scavenging ability. Acetylated chitosan has a low scavenging ability (between 13 and 30%) while the functionalization of chitosan with chelating agent improves hydroxyl scavenging ability: between 24 and 85% for Chito@DOTAGA and between 40 and 80% for Chito@DOTAGA@DFO. At low concentration (less than 2 g.L^−1^), the scavenging abilities of Chito@DOTAGA and Chito@DOTAGA@DFO are ordered according to their total chelation capacity (0.5 mmol.g^−1^ for Chito@DOTAGA@DFO vs 0.37 mmol.g^−1^ for Chito@DOTAGA). At high concentrations, the chelating agents are in excess compared to the iron concentration and the scavenging ability is similar for both functionalized chitosan. However, in comparison with the positive control, Trolox, all chitosan-based polymers retain their lower scavenging ability.

A second experiment with TiO_2_/UV hydroxyl generation was developed to evaluate the hydroxyl scavenging capacity of chelating polymers without the impact of iron (III) chelation, which can inhibit hydroxyl production. A similar quantification method was used to calculate the scavenging ability. Figure 4C confirmed the dose effect of chitosan polymers on hydroxyl scavenging (generated by TiO_2_/UV). As previously observed, functionalized chitosans have higher hydroxyl scavenging abilities compared to acetylated chitosan. Compared to hydroxyl generation by Fenton reaction, a reversed order was observed for the curves of Chito@DOTAGA and Chito@DOTAGA@DFO: for low concentration (less than 2 g.L^−1^), Chito@DOTAGA@DFO has a lower scavenging ability than the polymer without DFO, which could be explained by a slightly lower solubility of Chito@DOTAGA@DFO in 0.1 M phosphate buffer (pH 7.4) compared to Chito@DOTAGA.

### Iron extraction efficiency versus Deferiprone

**Figure 5:**
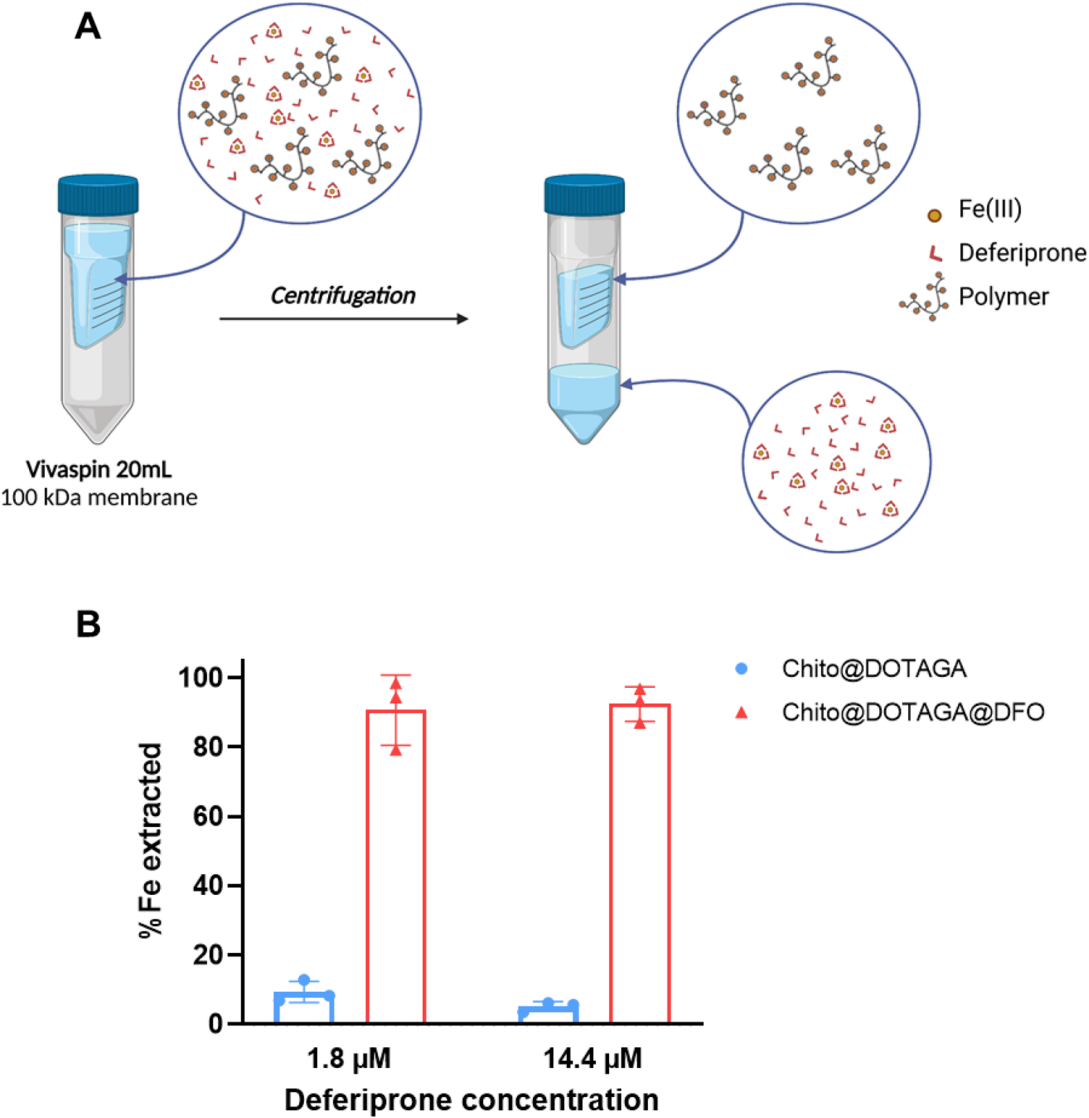
Iron extraction efficiency of Chito@DOTAGA@DFO in competition with Deferiprone. (**A**) Schematic representation of Vivaspin separation of polymer solutions (10 mg.L^−1^) and deferiprone (1.8 µM or 14.4 µM) in presence of iron(III) (0.45 µM) in 10 mM phosphate buffer, pH 7.7 (created with BioRender.com). (**B**) Percentage of iron extraction, calculated according to ^56^Fe concentration measured in undernatant by ICP-MS.

Iron extraction efficiency of Chito@DOTAGA and Chito@DOTAGA@DFO was further assessed in competition with deferiprone, a therapeutic iron chelating agent. The aim was to compare iron extraction efficiency of Chito@DOTAGA@DFO with a current treatment for iron overload. In the case of patients already treated with deferiprone and who require hemodialysis, it is also interesting to assess if Chito@DOTAGA@DFO would be able to extract iron from the therapeutic drug. This competition experiment was performed with a low iron concentration (0.45 µM) and an excess of deferiprone to avoid iron precipitation. Two concentrations of deferiprone were tested which corresponded to a small or large excess of deferiprone: 4 vs 32 molar equivalent compared to iron (deferiprone forms a 3:1 DFO:Fe^3+^ complex). In these conditions, the iron extraction efficiency of Chito@DOTAGA and Chito@DOTAGA@DFO at 10 mg.L^−1^ were tested. After one hour of incubation and centrifugation through 100 kDa Vivaspin membrane (Fig. 5A), the iron extraction efficiency was calculated from iron concentration in undernatant. In reference sample without the polymer, a similar concentration of iron is found in undernatant compared to initial solution which shows that deferiprone and deferiprone/iron complex are not retained in the supernatant (see Table S2 in Supporting Info). Figure 5B shows that at a low deferiprone concentration, Chito@DOTAGA is able to extract around 9.3% (Relative Standard Deviation (RSD): 3.1%) of the initial iron whereas Chito@DOTAGA@DFO can extract 90.7% (RSD: 10.1%). Additionally, in the presence of a large excess of deferiprone, Chito@DOTAGA is no longer able to extract iron while Chito@DOTAGA@DFO displays a similar extraction efficiency as the previous experiment (92.4%, RSD: 5.0%). Again, this increased extraction efficiency can be explained by the larger complexation constant of DFO compared to DOTA and Deferiprone (Table 1).

**Table 1:** Complexation constant of DOTA, Deferiprone and DFO in literature.

Chelator	DOTA	Deferiprone	DFO
Type of complex with Fe(III)	${\rm M}^{3+} + {\rm L}^{4-} \rightarrow {\rm ML}^{-}$	${\rm M}^{3+} + 3{\rm L}^{-} \rightarrow {\rm ML}_{3}$	${\rm M}^{3+} + {\rm LH}^{2-} \rightarrow {\rm (MHL)}^{+}$
Log Kc	29.4 25 °C	36.7 μ = 1.0 M, 25 °C	41.8 μ = 1.0 M, 25 °C
Reference	26	32	25

### Ex vivo efficacy of iron extraction by hemodialysis

**Figure 6:**
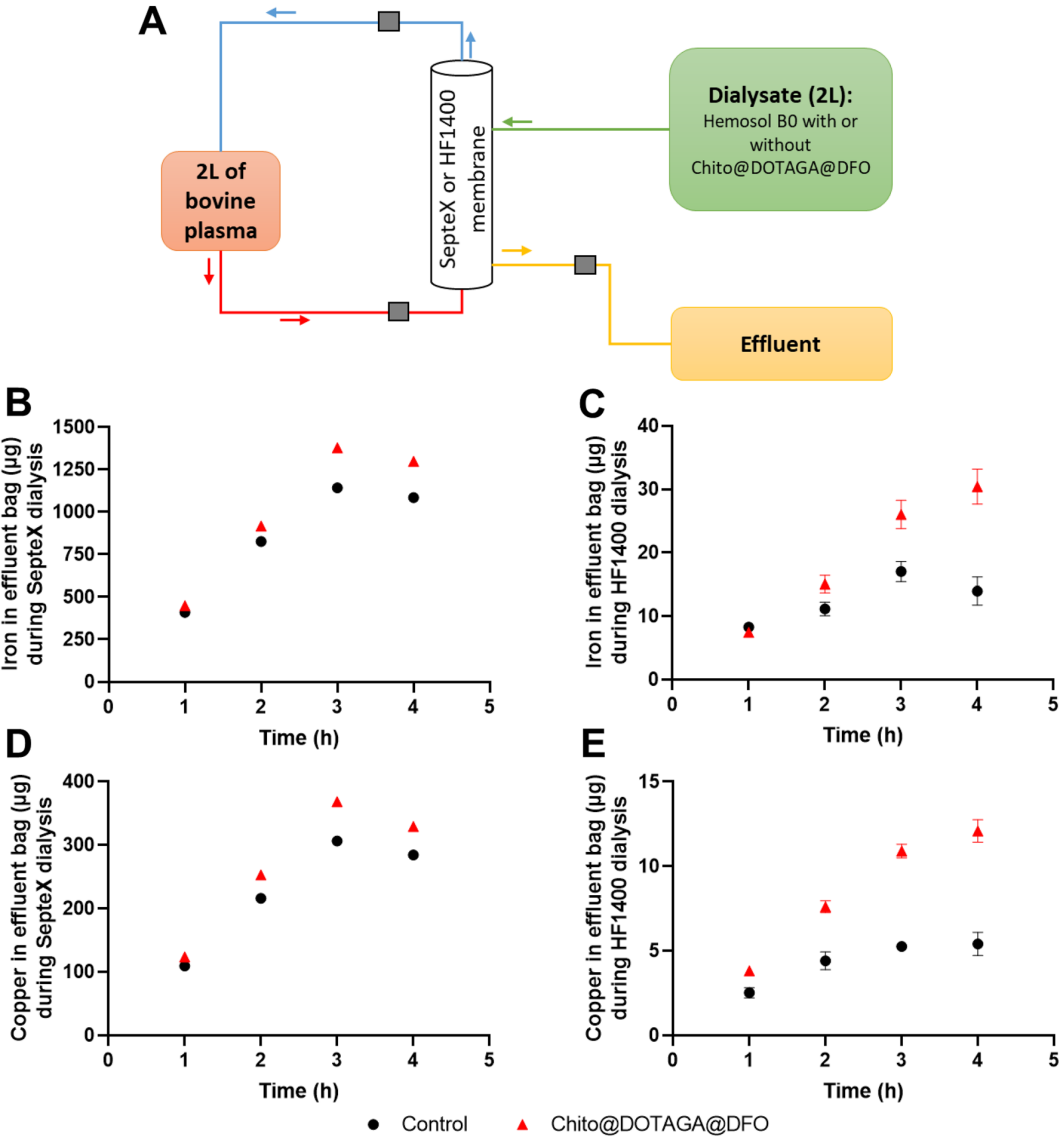
Ex vivo experiment for the quantitative assessment of metal extraction during hemodialysis with Chito@DOTAGA@DFO (red triangle) or Hemosol B0 (control, black circle). (**A**) Schematic representation of the experimental design. Evolution of iron amount (in µg) in effluent bag during dialysis with (**B**) SepteX and (**C**) HF1400 membrane. Evolution of copper amount (in µg) in effluent bag during dialysis with (**D**) SepteX and (**E**) HF1400 membrane. Standard error represents the variability among metal isotope (^63^Cu and ^65^Cu, ^56^Fe and ^54^Fe) sampled in duplicate. Errors bars are hidden by the symbol in figures (**B**) and (**D**) but standard deviations are available in supporting information.

The feasibility and iron extraction efficiency of hemodialysis with the Chito@DOTAGA@DFO polymer was assessed by an ex vivo experiment using bovine plasma. The addition of a Chito@DOTAGA@DFO solution into the Hemosol B0 bag results in a slightly yellow, clear and homogenous solution at 0.9 g L^−1^ of polymer. Moreover, the pH, osmolarity, and viscosity were stable throughout the experiment and non-significantly different from the Hemosol B0 alone (see Table S4). Dialysis pressure parameters (monitored by the Prismaflex monitor at the blood entry, the filter, the blood return, and the effluent, see Fig. 6A) were stable and within the normal range during the 4 h dialysis session with both the HF1400 (High flux, cut-off: 15–20 kDa) and the SepteX (high cut-off inferior to 45 kDa) dialyzer. The CVVHD session performed with the high cut-off membrane in the presence of Chito@DOTAGA@DFO leads to 1.3 mg of iron extracted (effluent bag, 4 h) versus 1.1 mg with the Hemosol B0 alone, corresponding to a 19% improvement of iron extraction (Fig. 6B). Similarly, copper extraction was increased by 14% when Chito@DOTAGA@DFO was added into the dialysate (330 µg versus 280 µg for control experiment, see Fig. 6D). Due to a lower cut-off which completely prevents the filtration of blood proteins and enzymes that can bind metals (including hemoglobin in our case), metallic extraction is limited with standard HF1400 dialyzer. Nevertheless, in these conditions, the addition of Chito@DOTAGA@DFO within the dialysate increases both iron and copper extraction by a factor 2.2 (30.4 µg vs 13.9 µg for iron and 12.1 µg vs 5.4 µg for copper, Fig. 6C and E).

## Discussion and conclusion

For pathologies characterized by iron overload, such as secondary hemochromatosis, metallic extraction is the cornerstone of disease management. Chelation therapy is currently the standard of care for these pathologies but does not seem to be adapted to the rapid metal liberation that can occur in patients in intensive care units (ICU) due to organ failure. The combination of a chelating polymer with hemodialysis, already implemented in ICU^33^, is a promising solution to improve metallic extraction of classical dialysis by overconcentration of the metal within the dialysate that cannot go back to the circulating blood due to the large size of the polymer and the cut-off of the membrane. In classical hemodialysis, metal extraction is driven by diffusion of metal ions through the membrane, and extraction is limited by the diffusion equilibrium. With the addition of a chelating polymer in the dialysate, the diffusion equilibrium will be shifted due to metal complexation by the polymer, which reduces the free metal concentration in solution.

Chitosan has been chosen for this application because (1) it is biocompatible, (2) it can easily be modified by functionalization of its primary amines, (3) it is soluble at physiological pH after partial acetylation (DA ~ 30–40%) and DOTAGA grafting, (4) it exhibits a sufficiently large molecular mass suppress as to prevent its passage through the dialysis membrane and (5) it is easily available and affordable in medical grade quality which is advantageous for a future industrialization^34–36^. Although chitosan has the ability to complex metals with the amino groups^37,38^, chelating agents were grafted to obtain an efficient extraction system. DOTA (1,4,7,10-tetraazacyclododecane-1,4,7,10-tetraacetic acid) was chosen as a chelating agent for this application due to its large complexation constants for Cu^2+^ and Fe^3+^ (log β = 22.4 and 29.4 respectively)^26^. To improve iron extraction, DFO (*N*-[5-[[4-[5-[acetyl(hydroxy)amino]pentylamino]-4-oxobutanoyl]-hydroxyamino]pentyl]-*N*′-(5-aminopentyl)-*N*′-hydroxybutanediamide) was chosen as a second chelating agent due to its higher complexation constant for Fe^3+^ (log β = 41.8)^25^. The chitosan backbone could also participate in the metal chelation thanks to its free amine groups^38^. A double-grafted polymer with DOTAGA and DFO (Chito@DOTAGA@DFO) was developed instead of a DFO-grafted polymer (Chito@DFO) due to its improved solubility at physiological pH (which is necessary for dilution in dialysate and for local administration) and its ability to chelate other redox active metals that can also be involved in the production of reactive oxygen species like copper. The direct grafting of p-NCS-Bz-DFO onto chitosan required a higher chitosan acetylation rate (x = 0.41) to achieve sufficient solubility at physiological pH and thus resulted in a lower DFO grafting rate (z = 0.027). In our case, Chito@DOTAGA@DFO displays high solubility at 1 g.L^−1^ and can chelate 0.53 mM of metals providing a maximum extraction capacity of 30 mg of iron per gram of polymer.

Antioxidant properties of functionalized chitosans were evaluated by classical in vitro tests. Many in vitro and in vivo studies have already shown that chitosans exhibit moderate redox-regulatory activity due to its inhibition of ROS production and prevention of lipid oxidation^39^. Several studies have highlighted that the degree of acetylation (DA) and molecular weight of the chitosan can drastically affect their antioxidant activity^31,40,41^. In literature, the radical scavenging mechanism of chitosan is thought to rely on radical interaction with hydroxyl and amine groups^31^. Poor solubility and intra- and inter-molecular interactions, which are influenced by degree of acetylation and molecular weight, can thus reduce the availability of the redox-active groups and have an impact on its antioxidant activity^39^. In addition, deferoxamine also exhibits antioxidant properties by scavenging of superoxide, alkoxyl and peroxyl radicals, independently of its iron chelating ability^42,43^. Here, functionalization of chitosan with both DOTAGA and DFO showed improved antioxidant properties in commonly used DPPH assay with a large range of polymer concentrations (from 0.1 to 10 g L^−1^). This improvement is mainly due to the DFO grafting since Chito@DOTAGA shows similar DPPH scavenging ability as acetylated chitosan. Chitosan with an acetylation degree around 30% and a molecular weight of 2.6 10^5^ g.mol^−1^ showed a scavenging ability around 10% at 1 g.L^−1^, which is consistent with DPPH scavenging ability measured by Avelelas et al.^31^ on a similar molecular weight water-soluble chitosan with slightly higher acetylation degree (15% SA at 1 g.L^−1^ for a chitosan with DD = 55% and Mw = 2.79 10^5^ g.mol^−1^). Furthermore, Chito@DOTAGA@DFO was able to reduce DPPH with a high scavenging ability (90–95%) similar to that of the ascorbic acid reference which was already observed for deferoxamine by Adjimani et al*.* in a similar DPPH assay^44^.

Regarding hydroxyl scavenging, the two methods of hydroxyl generation highlight the antioxidant ability of chitosan-based polymers with or without its metal chelating effect. By Fenton generation, functionalized chelating polymers Chito@DOTAGA@DFO and Chito@DOTAGA showed a significantly improved hydroxyl scavenging ability compared to acetylated chitosan. Furthermore, Chito@DOTAGA@DFO showed a higher scavenging ability compared to Chito@DOTAGA which can be explained by the larger complexation constant of DFO for Fe(III) than DOTA and a higher total grafting rate of the chelating agents (0.130 vs 0.077). However, at 5 g L^−1^, Chito@DOTAGA and Chito@DOTAGA@DFO have similar hydroxyl scavenging ability, probably due to reduced solubility and higher viscosity of Chito@DOTAGA@DFO at this concentration. Several studies indeed emphasized that chitosan antioxidant activity can be limited by its solubility, viscosity, and the strong inter- and intra-molecular hydrogen bonds^39,45^. In this work, an assay to quantify hydroxyl scavenging ability of chitosan-based polymer independently of their chelating properties using TiO_2_ photo-catalysis was also developed. With this method, both chitosan and functionalized chitosans were shown to directly scavenge hydroxyl radical, with a significant improvement of scavenging ability for functionalized chitosans that could be explained by an improved solubility. Here, the hydroxyl scavenging of chitosan relies on the direct reaction of OH^•^ with hydrogen atoms of chitosan to form a more stable radical, as hypothesized by Li et al.^45^.

Finally, the iron extraction efficiency of the chelating polymers was assessed in competition with deferiprone, a therapeutic iron chelating agent. For this competition experiment, a low iron concentration (0.45 µM) was chosen as a good representation of the level of labile plasmatic iron in healthy patients. In literature, labile plasmatic iron concentration in healthy patients are reported between 0.2 and 1.5 µM^46^. Thus, we demonstrated the extraction efficiency of Chito@DOTAGA@DFO at very low iron concentration in competition with a strong iron chelating agent. At the highest tested deferiprone concentration, deferiprone and Chito@DOTAGA@DFO are both found in large excess compared to iron concentration (around 11 times given that deferiprone forms a 3:1 complex with Fe^3+^), and the high extraction percentage (> 90%) measured for Chito@DOTAGA@DFO is due to the largest complexation constant of DFO compared to deferiprone (log β = 41.8 vs 36.7 for deferiprone)^25,32^.

The feasibility to perform hemodialysis with Chito@DOTAGA@DFO was verified during an *ex vivo* experiment on bovine plasma. The plasma chosen had a high percentage of hematocrit and the presence of hemoglobin greatly increases the total iron content of the plasma (around 2000 µg.L^−1^), which can simulate an iron overload situation. In healthy patients, the typical plasmatic iron concentration is around 20 µM, i.e. around 1100 µg.L^−1^^3^. Here, Chito@DOTAGA@DFO in the dialysate increases the iron extraction by a factor 2.2 with standard HF1400 dialyzer despite a limited metallic extraction due to a lower cut-off that completely prevents blood proteins and enzymes containing metals (including hemoglobin) to pass through the membrane. However, during the 4 h dialysis session with SepteX dialyzer (high cut-off), 1.3 mg of iron were extracted in presence of Chito@DOTAGA@DFO, which corresponds to an improvement of 19% compared to classic hemodialysis with Hemosol B0. Copper extraction was also improved with both dialyzers compared to classical dialysis fluid. Since copper can also catalyze the production of reactive oxygen species through the Fenton reaction, a combined extraction of copper and iron could considerably reduce the oxidative damages caused by these two metals.

In conclusion, a water-soluble chitosan-based polymer with chelating and antioxidant properties was synthetized. The feasibility to combine this polymer with conventional dialysis techniques for iron extraction was demonstrated on an iron-laden bovine plasma, and an improvement of iron extraction compared to conventional hemodialysis was observed. All of these results provide evidence that this versatile polymer can extract iron in physiological conditions and are encouraging for future applications in intensive care units or for localized administration. All of these conclusions should to be confirmed in future in vivo experiments, initially by assessing the safety and efficacy of the chelating polymer within hemodialysis on large animals or by a local administration on small animals.

## Material and methods

### Synthesis of Chito@DOTAGA@DFO

*Chemicals:* Freeze-dried Chito@DOTAGA was provided by MexBrain (Villeurbanne, France). It has been synthetized as described in Natuzzi et al.^23^. Briefly, the starting material is a medical grade chitosan with a low degree of acetylation (4.5% ± 0.5%, determined by ^1^H NMR spectroscopy by the Hirai method)^24^ and with Mw = 2.583 10^5^ g.mol^−1^ (determined by size exclusion chromatography coupled with refractive index and multi-angle laser light scattering measurements)^47^. Acetylation of this initial chitosan was performed by addition of pure acetic anhydride in a water/1,2-propanediol mixture chitosan solution. After this first step, re-acetylated chitosan (Reac-Chito) was obtained and a sample of the reaction mixture was purified by tangential filtration and freeze-dried for assessment of acetylation degree by ^1^H NMR spectroscopy. Functionalization with DOTAGA was obtained by direct addition of DOTAGA anhydride (1,4,7,10-tetra-azacyclododecane-1-glutaric anhydride-4,7,10-triacetic acid) to the solution of Reac-Chito in water/1,2-propanediol. p-NCS-Bz-DFO (N1-hydroxy-N1-(5-(4-(hydroxy(5-(3-(4-isothiocyanatophenyl)thioureido)pentyl)amino)-4-oxobutanamido)pentyl)-N4-(5-(N-hydroxyacetamido)pentyl)succinamide) was furnished by Chematech (Dijon, France). 1,2-propanediol, DMSO and acetic anhydride were purchased from Sigma-Aldrich (Saint-Quentin-Fallavier, France). Glacial acetic acid (AnalR NormaPur) grade was furnished by VWR (Rosny-sous-Bois, France).

#### Grafting of DFO

20 g of freeze-dried Chito@DOTAGA were dissolved in 2 L of ultra-pure water. This solution was stirred at room temperature until complete dissolution of the polymer. The pH was measured to be 6.3. A second solution containing p-NCS-Bz-DFO was then prepared by dissolving 3.8 g of p-NCS-Bz-DFO in 380 mL of DMSO. The solution was stirred at room temperature for at least 30 min. Then the solution of Chito@DOTAGA in water was poured in a 10 L reactor and 80 mL of ultrapure water were added. Then, 1.2 L of 1,2-propanediol was added under stirring and at controlled temperature (30 °C). 380 mL of DMSO were then added slowly into the reactor. The mixture was stirred at 30 °C for 1 h, before slow addition of the solution of p-NCS-Bz-DFO in the reactor using a peristaltic pump at a flow rate of 400 µL min^−1^. The reactor was maintained at 30 °C during the reaction and stirred for 16.5 h. After this, the synthetized product was purified by tangential filtration using a 100 kDa cut-off membrane (Sartocon Slice 200, PES membrane) with a Sartoflow Advanced apparatus. Briefly, the mixture was diluted by 5 in acetic acid (0.1 M) then concentrated to reach a dilution factor of 2 compared to the initial volume. Then, the product was purified using acetic acid 0.1 M then ultra-pure water and re-concentrated to the initial volume.

### Characterization of DFO grafting by UV–Vis titration

The amount of DFO grafted was determined by titration with Fe(III) by measuring the absorption at 429 nm (maximum wavelength of DFO-Fe(III) complex)^27^. Polymer is first dissolved in water at 5 g L^−1^. Then various solutions of 0.1 g L^−1^ polymer in ammonium acetate (0.1 M)/acetic acid (0.1 M) buffer were prepared with different concentrations of FeCl_3_, ranging from 0 to 80 µM. The absorption at 429 nm was then plotted versus the iron content in solution, calculated in mmol per gram of polymer. After linear regression, the iron chelation capacity by DFO is determined as the concentration at the observed change of slope.

### Characterization of DFO grafting by ^1^H NMR

^1^H NMR spectra were performed at the Polymer NMR Platform of Institut de Chimie de Lyon (Axel’One Campus-Lyon) on a Bruker Avance III spectrometer operating at 400.1 MHz for ^1^H observation and equipped with a 5 mm BBFO + probe. Samples were dissolved in D_2_O at 5 g.L^−1^. ^1^H spectra were recorded with a 30° flip angle, 6.0 s recycle delay and 512 scans at 343 K. TopSpin software was used for processing. Baseline correction, phase adjustment, and integration were processed manually.

### Assessment of antioxidant power

*Chemicals:* 2,2-diphenyl 1-picrylhydrazyle (DPPH, CAS 1898-66-4), L-ascorbic acid (99%, CAS 50-81-7), glacial acetic acid (99.5%, CAS 64-19-7), methanol (98.5%, CAS 67-56-1), EDTA calcium disodium salt (≥ 97%, CAS 62-33-9), iron sulfate heptahydrate (≥ 99.5%, CAS 7782-63-0), (±)-6-Hydroxy-2,5,7,8-tetramethylchromane-2-carboxylic acid (Trolox, 97%, CAS 53188-07-1), HEPES (≥ 99.5%, CAS 7365–4569), H_2_O_2_ 30% (v/v) (Reagent grade, CAS 7722-84-1), sodium benzoate (≥ 99.5%, CAS 532-32-1), sodium phosphate dibasic (≥ 99.0%, CAS 7558-79-4) and potassium phosphate monobasic (≥ 99.0%, CAS 7778-77-0) were purchased from Sigma-Aldrich (Saint-Quentin-Fallavier, France).

#### Preparation of antioxidant solutions

Chito@DFO (re-acetylated chitosan only grafted with DFO, x = 0.41 and z = 0.027) was synthetized as described in Supporting Info. Briefly, acetylation of chitosan was performed by addition of pure acetic anhydride in a water/1,2-propanediol mixture. Functionalization with DFO was obtained by slow addition of a solution of p-NCS-Bz-DFO in DMSO to the previous solution in a water/1,2-propanediol/DMSO mixture. Re-acetylated chitosan (Reac-Chito, x = 0.29) was purified following the re-acetylation step of Chito@DOTAGA synthesis as previously described. For DPPH assay, a polymer stock solution was prepared at 20 g L^−1^ then this solution was diluted at various concentrations and the pH was adjusted to 4 with acetic acid. Ascorbic acid solution was prepared similarly.

#### DPPH scavenging ability

The determination of 2,2-diphenyl 1-picrylhydrazyle (DPPH) scavenging by chitosan-based polymers was adapted from a method previously described by Avelelas et al.^31^. Samples were prepared by mixing 1 mL of DPPH solution (0.2 mM in methanol) and 1 mL of antioxidant solution previously prepared. All samples were prepared in triplicates, protected from light and stirred. Absorbance at 517 nm is measured after 1 h and 19 h with a Cary 50 Scan UV–visible spectrophotometer using plastic cuvettes with optical path length of 10 mm. Scavenging ability (SA) was calculated as follow:

$$SA\left( \% \right) = \left( {1 - \frac{{Abs\left( {sample} \right)}}{{Abs\left( {control} \right)}}} \right) \times 100$$

where *Abs(sample)* is the absorbance measured for samples containing antioxidants and *Abs(control)* the absorbance of control sample with only DPPH in the mixture methanol/water at pH 4 after pH adjustment with acetic acid.

#### Fenton-generated hydroxyl radical scavenging

Hydroxyl radical scavenging was determined with a method inspired by Feng et al.^40^. A solution of FeSO_4_ at 5 mM was prepared in 10 mM EDTA solution (pH adjusted to 3). 0.6 mL of this solution was added dropwise to 4.2 mL of antioxidant solution (chitosan-based polymers or Trolox as positive control) previously solubilized in 0.1 M HEPES buffer (pH 7.4). Then 0.6 mL of 10 mM sodium benzoate (NaBz) and 0.6 mL of 10 mM H_2_O_2_ were added and the sample was stirred at 37 °C for 2 h. After centrifugation (4000 rpm, 15 min), fluorescence from the supernatant was analyzed with a Cary Eclipse Fluorescence Spectrophotometer (excitation at 310 nm, emission at 407 nm) using plastic cuvettes with optical path length of 10 mm. Fluorescence intensity was also measured for a blank sample containing only 1 mM NaBz in 0.1 M HEPES. Scavenging ability (SA) was calculated as follow:

$$SA\left( \% \right) = \left( {1 - \frac{{I_{F} \left( {sample} \right)}}{{I_{F} \left( {control} \right)}}} \right) \times 100$$

where ${I}_{F}\left(sample\right)$ is fluorescence intensity (310/407 nm) of samples containing antioxidants and ${I}_{F}(control)$ is fluorescence intensity of negative control (without antioxidants).

*TiO*_*2*_*/UV-generated hydroxyl radical scavenging:* The assay was based on hydroxyl radical measuring method developed by Xiang et al.^48^. 5 mg of TiO_2_ (85% anatase, 15% rutile) were introduced in vials and 4.8 mL of antioxidant solution (chitosan-based polymers or Trolox) in 0.1 M phosphate buffer (pH 7.4) were added. Under stirring, 0.6 mL of 10 mM sodium benzoate (NaBz) and 0.6 mL of 30% w/w H_2_O_2_ were added to the sample. Samples are placed under UV-radiation at 375 nm and stirred for 2 h. The light source (ThorLabs M375L3) was placed 10 cm above sample surface. After centrifugation (4000 rpm, 10 min), the fluorescence intensity from supernatant was analyzed with a Cary Eclipse Fluorescence Spectrophotometer (excitation at 310 nm, emission at 407 nm) using plastic cuvettes with optical path length of 10 mm. Fluorescence intensity was also measured for a blank sample containing only 1 mM NaBz in 0.1 M phosphate buffer. Scavenging ability (SA) was calculated similarly to Fenton-generated hydroxyl radical scavenging.

### Evaluation of iron extraction in competition with Deferiprone

#### Chemicals

Standard iron solutions for ICP-MS were provided by SCP-Science (ICP Standard 50,000 µg mL^−1^, 140-041-265 and ICP Standard 1000 µg mL^−1^, 140-051-260). Deferiprone (purity 98%, CAS 30652-11-0), sodium phosphate dibasic (ACS reagent ≥ 99.0%, CAS 7558-79-4) and potassium phosphate monobasic (Reagent Plus ≥ 99.0%, CAS 7778-77-0) were purchased from Sigma-Aldrich (Saint-Quentin-Fallavier, France).

#### Preparation of stock solutions

Freeze-dried Chito@DOTAGA and Chito@DOTAGA@DFO were both dissolved in ultra-pure water at a concentration of 1 g L^−1^. A solution of Fe(III) at 1 mg.L^−1^ in 10^–2^ M HCl was prepared by dilution of the standard iron solution at 50,000 mg.L^−1^. Deferiprone was first solubilized at 10 g.L^−1^ in ultra-pure water then diluted to reach a concentration of 10 mg L^−1^. Phosphate buffer (10 mM, pH 7.7) was prepared from sodium phosphate dibasic and potassium phosphate monobasic dissolved in ultra-pure water.

#### Experimental protocol

From all previously described stock solutions, samples (20 mL) were prepared in 10 mM phosphate buffer with the following concentrations: 0.45 µM of iron (III), 1.8 µM or 14.4 µM of deferiprone, 10 mg.L^−1^ of polymer (Table 2). Reference samples were also prepared with only polymer in phosphate buffer or with only iron(III) and deferiprone but without polymer. Samples were stirred at room temperature for one hour, then centrifuged using 20 mL Vivaspin with a 100 kDa PES cut-off membrane. The principle of the experiment is described in Fig. 5A. Briefly, due to the larger size of the polymer compared to membrane cut-off, the polymer cannot go through the membrane whereas small molecules, such as deferiprone, will cross the membrane. Centrifugation was performed at 2000 rpm until around 2 mL of supernatant was recovered.

**Table 2:** Summary of sample compositions for iron extraction in competition with deferiprone. All samples were prepared in 10 mM phosphate buffer (pH 7.7).

Sample	Polymer	Total chelator concentration (µM)	Iron concentration (µM)	Deferiprone concentration (µM)
1	Chito@DOTAGA	3.3	N.A.	N.A.
2	Chito@DOTAGA@DFO	5.0	N.A.	N.A.
3	N.A.	N.A.	0.45	1.80
4	N.A.	N.A.	0.45	14.4
5	Chito@DOTAGA	3.3	0.45	1.80
6	Chito@DOTAGA	3.3	0.45	14.4
7	Chito@DOTAGA@DFO	5.0	0.45	1.80
8	Chito@DOTAGA@DFO	5.0	0.45	14.4

#### Iron quantification by ICP-MS

The ^56^Fe content in the initial solution (before polymer addition), in the undernatant and in the supernatant after centrifugation were measured by ICP-MS. ICP-MS analysis was performed using a Perkin Elmer NexION2000 equipped with Syngistix software (Licensed software by Perkin Elmer. Version: 2.3 (Build 2.3.7916.0)) and a ESI SC-FAST Sample Introduction in Kinetic Energy Discrimination (KED) mode. The samples were prepared by dilution in an aqueous solution of 1% (v/v) HNO_3_.

### Ex vivo efficacy of iron extraction by hemodialysis

#### Chemicals

Hemosol B0 dialysate solution was produced by Baxter. The standard Cu and Fe solutions for the ICP-MS are provided by SCP-Science: ICP Standard Cu 1000 µg.mL^−1^ (SCP Science, 14-051-290) and ICP Standard Fe 1000 µg.mL^−1^ (SCP Science, 14-051-260).

#### Preparation of Chito@DOTAGA@DFO and Hemosol B0 dialysate

2.2 L or 1.8 L of reconstituted Hemosol B0 were manually withdrawn from a 5 L Hemosol B0 bag and introduced into a clean dialysis bag using a peristaltic pump. 2.2 L of Hemosol B0 were used to conduct the experiment without polymer and 1800 mL of Hemosol B0 were completed with 400 mL of sterile liquid formulation of Chito@DOTAGA@DFO (5 g.L^−1^ in 0.7% NaCl solution) to obtain a final concentration of 0.9 g.L^−1^ of Chito@DOTAGA@DFO.

#### Preparation of bovine plasma bag

Four 500-mL bottles of frozen bovine plasma with sodium citrate (EUROBIO SCIENTIFIC, reference S0260-500, hematocrit 18.72 mg/100 mL) were placed overnight at 4 °C to thaw. In the morning, the 500-mL bottles of bovine plasma were placed in a dry oven at 37 °C until temperature stabilization. The bovine plasma was then filtered on a wide mesh (1.5 mm) to remove protein clumps and transferred in a 5 L effluent bag using a peristaltic pump. 100 µL of heparin sodium (Heparin Choay®, 5000 U.I./mL, Sanofi) was added to the plasma bag to prevent coagulation.

#### Experimental protocol

Dialysis protocol is illustrated in Fig. 6A using a Prismaflex monitor and two types of dialyzers: HF1400 set (High-flux membrane, 15–20 kDa, 1.4 m^2^) or SepteX set (High cut-off, < 45 kDa, 1.1 m^2^). The dialysate flow rate was fixed at 500 mL.h^−1^ and the blood flow rate was maintained at 100 mL.min^−1^. For each dialyzer types, two dialysis were performed: reference experiment with Hemosol B0 alone or experiment with Hemosol B0 and Chito@DOTAGA@DFO. The duration of all dialysis experiments was 4 h. Sampling for iron quantification was performed at time zero from the dialysate and every hour from the effluent bag. All samples were obtained in duplicates.

#### Metal quantification by ICP-MS

ICP-MS analysis was performed using a Perkin Elmer NexION2000 equipped with Syngistix software (Licensed software by Perkin Elmer. Version: 2.3 (Build 2.3.7916.0)) and an ESI SC-FAST Sample Introduction in Kinetic Energy Discrimination (KED) mode. The samples were prepared by dilution in an aqueous solution of 1% (v/v) HNO_3_. 250 µL of dialysate samples were diluted in 4.7 mL of 1% (v/v) HNO_3_ solution and 50 µL of 200 µg.L^−1^ indium solution was added as internal standard.

#### Data analysis

The metallic extraction from the effluent bag at each time point was corrected with the initial metallic content of Chito@DOTAGA@DFO or Hemosol B0 in the dialysate bag at time zero (t0) as follow: effluent sample (ppb, Tx)−dialysate sample (ppb, t0) = effluent sample corrected (ppb, Tx). The metallic content (µg) was obtained by multiplying the effluent sample concentration (corrected, ppb) by the real volume of dialysate (L) for the time point of interest (T1 hour: 0.5 L, T2 hours: 1 L, T3 hours: 1.5 L, T4 hours: 2L). Standard deviations of raw data were calculated from the four replicates (two isotopes (^63^Cu and ^65^Cu for copper, ^56^Fe and ^54^Fe for iron) sampled in duplicate). Standard deviations for corrected values were calculated as follow: ${RSD}_{Corrected values}=\sqrt{\frac{RSD1}{N1}+\frac{RSD2}{N2}}$ where RSD1 and RSD2 are the standard deviations of the two values that are subtracted and N1 and N2 are the number of replicates of the two values that are subtracted. RSD for metallic content (µg) were obtained by multiplying the sample concentration RSD (corrected, ppb) by the actual volume of dialysate (L) at the concerned time point. All calculations and data are detailed in Supporting Info.

## Supplementary Information

Supplementary Information.

## Data Availability

All datasets generated by ICP-MS analysis are available in Supporting Info. All datasets used during the current study are also available from the corresponding author on reasonable request.
