# Supplementary material for: Design of a water-soluble chitosan-based polymer with antioxidant and chelating properties for labile iron extraction
Source: Sci Rep. 2023 May 16;13:7920. doi: 10.1038/s41598-023-34251-3 (PMC10188602; doi:10.1038/s41598-023-34251-3)
Supplement: Supplementary file 1 — Supplementary Information. [file 41598_2023_34251_MOESM1_ESM.docx]

**Design of a water-soluble chitosan-based polymer with antioxidant and chelating properties for labile iron extraction**

**Coralie Grange^1,2^, Axel Aigle^1^, Victor Ehrlich^2^, Juan Felipe Salazar Ariza^2,3^, Thomas Brichart^1^, Fernande Da Cruz-Boisson^3^, Laurent David^3^, François Lux^2,4,*^_,_ Olivier Tillement^2^.**

^1^ MexBrain, 13 avenue Albert Einstein, Villeurbanne, France

^2^ Institut Lumière-Matière, UMR 5306, Université Lyon1-CNRS, Université de Lyon, Villeurbanne Cedex, France; Institut Universitaire de France, Paris, France.

^3^ Ingénierie des Matériaux Polymères, CNRS UMR 5223, Univ Claude Bernard Lyon 1, Institut national des Sciences Appliquées, Université Jean Monnet, Univ Lyon, 15 bd Latarjet, 69622 Villeurbanne, France.

^4^ Institut Universitaire de France (IUF), 75231 Paris, France.

* Correspondence and requests for materials should be addressed to Dr François Lux (email: francois.lux@univ-lyon1.fr)

**Characterization of Chito@DOTAGA@DFO.**


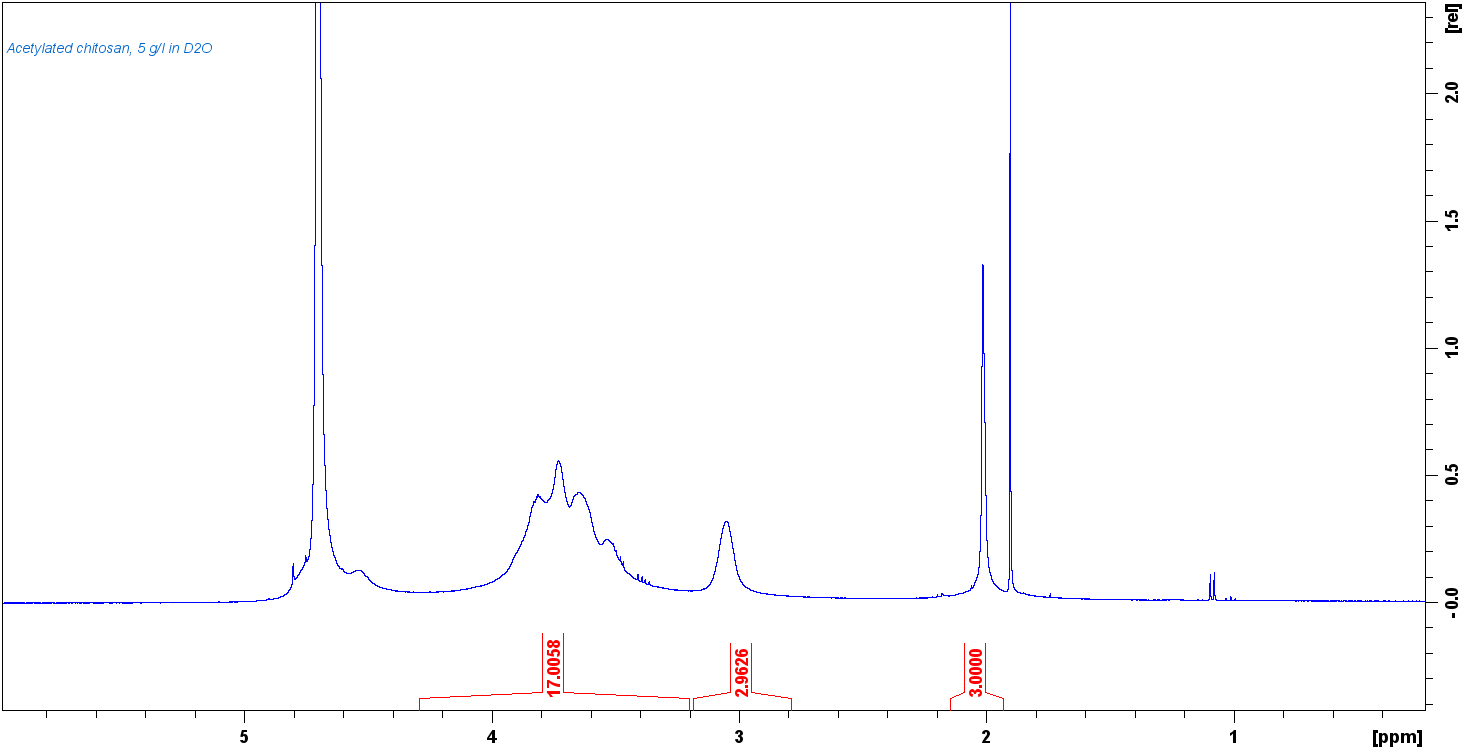
**Figure S1.** ^1^H NMR spectrum of reacetylated chitosan (29%) in D_2_O at 5 g.L^-1^. Peak at 1.9 ppm is attributed to residual acetate group from acetic acid and doublet at 1.1 ppm corresponds to residual 1,2-propanediol.

**Figure S2.** Dosage of a solution of 1 g.L^-1^ of Chito@DOTAGA by Cu(II) in acetate buffer (0.1 M ammonium acetate, 0.1 M acetic acid) using UV-Vis spectroscopy and plotting absorbance at 295 nm. DOTAGA grafting rate was determined to be y=0.077.

**Figure S3.** UV-Vis absorption spectrum of commercial DFO (DFO mesylate) at 0.2 mM with (black) or without addition of 0.2 mM Fe(III) (red).

**
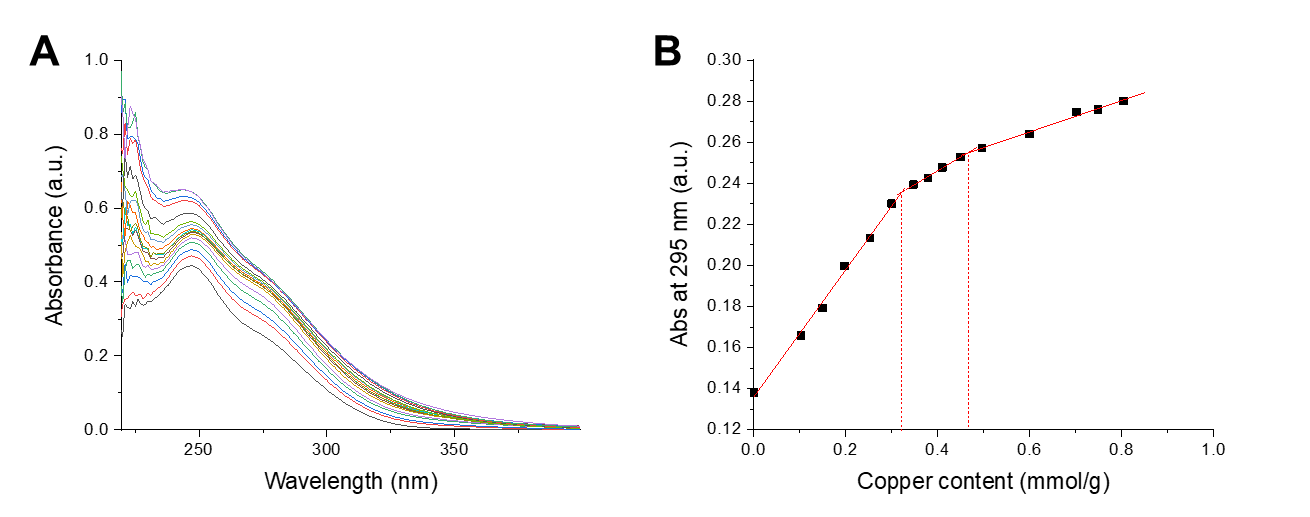
**

**Figure S4. (A)** Dosage of a solution of 0.1 g.L^-1^ of Chito@DOTAGA@DFO by Cu(II) in acetate buffer (0.1 M ammonium acetate, 0.1 M acetic acid) using UV-Vis spectroscopy and **(B)** plotting absorbance at 295 nm. The first slope discontinuity corresponds to DOTAGA saturation with Cu(II) (y=0.078).

**Computation of parameter *z* from UV data.**

The mass fraction of the DFO-grafted repeat unit, $w,$ is defined as

$$w\text{ }=\frac{{n_{DFO}\cdot M}_{z}}{m_{Chitosan}}= \frac{z\cdot M_{z}}{x\cdot M_{x}+y\cdot M_{y}+{z\cdot M}_{z}+{a\cdot M}_{a}}$$

Where $n_{DFO}$ is the mole number of DFO-grafted repeat unit, $m_{Chitosan}$ is the (dry) mass of dissolved Chito@DOTAGA@DFO in solution, $x$ is the molar fraction of N-acetyl glucosamine repeat units, $y$=$n_{DOTAGA}/n_{TOT}$ is the molar fraction of the DOTAGA-grafted repeat units, $z=n_{DFO}/n_{TOT}$ is the molar fraction of the DFO-grafted repeat units and $a$ is the molar fraction of the glucosamine repeat units. Accordingly, $M_{x}$ is defined as the molar mass of N-acetyl repeat units, $M_{y}$ is the molar mass of DOTAGA-grafted repeat units, $M_{z}$ is the molar mass of DFO-grafted repeat units and $M_{a}$ the molar mass of glucosamine residue.

Since $a=1-x-y-z$ the equation above becomes

$$w=\frac{z\cdot M_{z}}{x\cdot\left( M_{x}-M_{a} \right)+y\cdot\left( M_{y}-M_{a} \right)+z\cdot\left( M_{z}-M_{a} \right)+M_{a}}$$

Therefore

$$z\text{ }= \frac{w\cdot(x\cdot\left( M_{x}-M_{a} \right)+y\cdot\left( M_{y}-M_{a} \right)+M_{a})}{M_{z}-w\cdot\left( M_{z}-M_{a} \right)}$$

By computation,

$$M_{x}=203.19 g.{mol}^{-1} , M_{y}=619.62 g.{mol}^{-1}, {M_{z}=914.06 g.{mol}^{-1}, M}_{a}=161.16 g.{mol}^{-1}\text{}$$

Using $m_{TOT}$= 1 g of hydrated Chito@DOTAGA@DFO dissolved in solution, from the UV titration with Fe(III), we find:

$$n_{DFO}=0.200 mmol$$

In order to evaluate the mass fraction $w$, we evaluated a water content of $w$c = 8% w/w in the lyophilized Chito@DOTAGA@DFO powder by a Karl Fischer test. Then,

$$w\text{ }=\frac{{n_{DFO}\cdot M}_{z}}{m_{TOT}\cdot(1-wc)}$$

Hence, $w\text{ }=0.199$

From ^1^H-NMR, $x=0.29$ and from the UV-vis titration of Chito@DOTAGA with Cu(II), $y=0.077$.

This results in

$$z=0.054$$

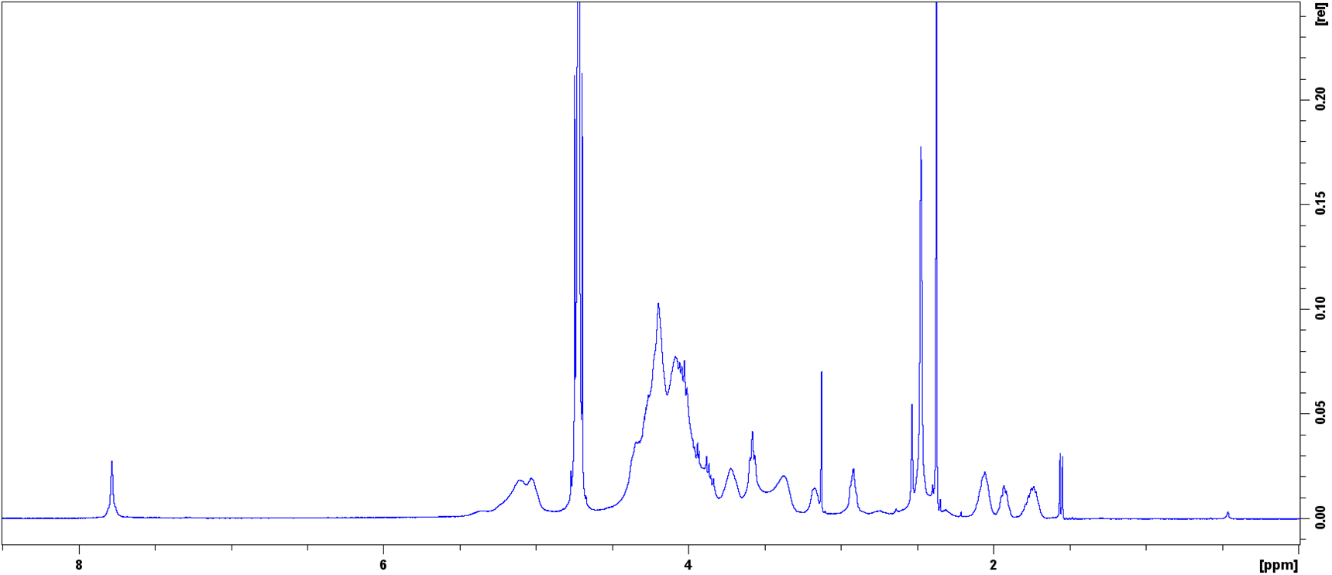


**Figure S5.** NMR ^1^H spectrum of Chito@DOTAGA@DFO in D_2_O at 5 g.L^-1^. Peak at 2.4 ppm is attributed to residual acetate groups from acetic acid and doublet at 1.6 ppm corresponds to residual 1,2-propanediol. Peak at 3.2 ppm corresponds to DMSO.

**Computation of parameter** $\boldsymbol{z}$ **from ^1^H NMR.**

DFO-grafting rate ($z$) can be obtained by ^1^H NMR through the ratio between the area of the peak at 7.8 ppm (attributed to aromatic protons H_n_ and H_n’_ from p-NCS-Bz-DFO, see Figure S6 and Table S1) over the area of the signals in the region 3.3-4.6 ppm following the equation:

$$\frac{{Area}_{3.3-4.6ppm}}{{Area}_{7.8ppm}}=\frac{6 + 25y + 12z}{4z}$$

From ^1^H-NMR of acetylated chitosan (Figure S1), $x=0.29$ and from the UV-vis titration of Chito@DOTAGA@DFO with Cu(II), $y=0.078$, this results in $z=0.046\pm0.002$.

It is also possible to determine $z$ through the ratio between the area of the three peaks from 1.6 to 2.2 ppm (attributed to the 18 protons from CH_2_ groups of p-NCS-Bz-DFO) over the area in the region 3.3‑4.6 ppm following the equation:

$$\frac{{Area}_{3.3-4.6ppm}}{{Area}_{1.6-2.2ppm}}=\frac{6 + 25y + 12z}{18z}$$

Similarly, this results in $z=0.044\pm0.002$.

The slight difference with $z$ value calculated by UV-Vis titration with Fe(III) could be explained by the difficulties to integrate the mass of peaks between 3.3 and 4.6 ppm (due to a signal that does not clearly return to baseline). The signal was thus integrated several times and DFO-grafting rate was calculated for each integration to obtain a mean value with standard deviation. In addition, the presence of residual solvents visible in the ^1^H NMR spectrum of Chito@DOTAGA@DFO can also explained the difference as it increases the area in the region 3.3-4.6 ppm thus leading to underestimated value of $z$ by ^1^H NMR.

| **Chemical shift (ppm)** | **Integration** | **Attribution** |
| --- | --- | --- |
| 7.76 | 4z | H_n_ et H_n’_ |
| 4.89-5.50 | 1 | H_1_ |
| 4.55-3.27 | 6 + 12z + 25y | H_2_, H_3_, H_4_, H_5_, H_6_, H_7_, H_a_, H_e_, H_l_, H_h_  + 25 protons from DOTAGA (in red) |
| 3.15 | 4z | H_f_ |
| 2.90 | 4z | H_g_ |
| 2.51 | 3z | H_m_ |
| 2.45 | 3x + 2y | H_ac_, H_β_ |
| 2.04 | 18z | H_b_, H_c_, H_d_, H_k,_ H_i,_ H_j_ |
| 1.91 |  |  |
| 1.72 |  |  |

**Table S1:** Attribution of ^1^H NMR signals of Chito@DOTAGA@DFO (x: acetylation degree, y: DOTAGA grafting, z: DFO grafting)

**
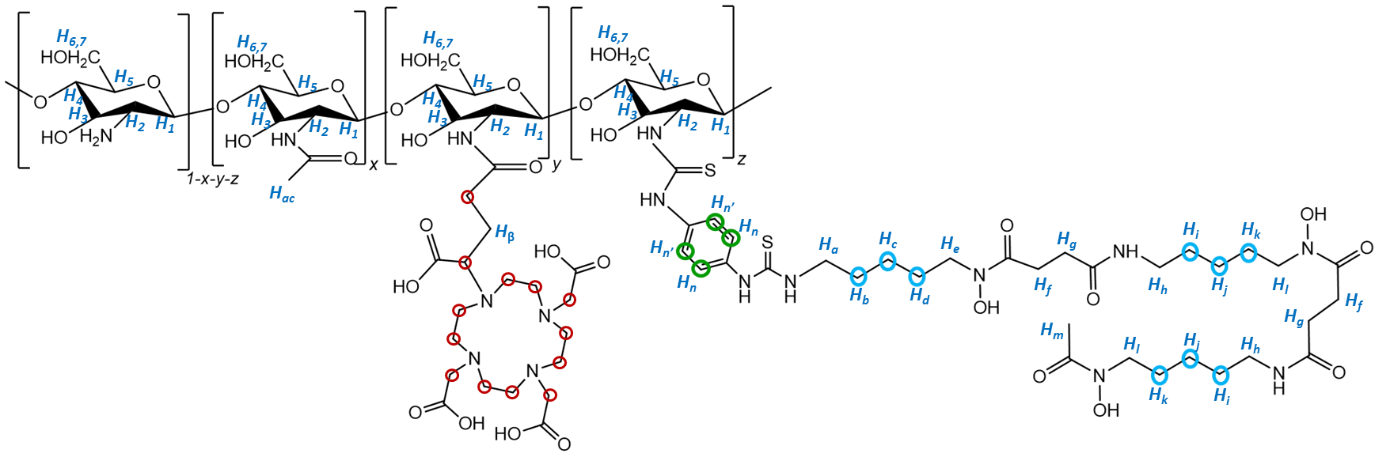
**

**Figure S6:** Labelling for attribution of ^1^H NMR signal of Chito@DOTAGA@DFO

**Antioxidant power of Chito@DOTAGA@DFO.**

**Synthesis of Chito@DFO:**

2.5 g of chitosan were dissolved in 170 mL of ultra-pure water and 2.3 mL of glacial acetic acid. Once solubilized, 50 mL of propanediol were added to the solution, the solution was stirred for 1 hour. 0.811 mL of acetic anhydride were added to 25 mL of propanediol. This solution was slowly added to the chitosan solution. The mixture was then stirred for at least 4 hours. 43 mL of ultra-pure water and 40 mL of NaOH 1 M were added to the previous solution. pH was measured at 5.8. 76 mL of propanediol and 35 mL of DMSO were added and the mixture was stirred at 30°C for 30 minutes. A solution of p‑NCS-Bz-DFO was prepared by dissolving 585 mg of p-NCS-Bz-DFO in 58.5 mL of DMSO. Once solubilized, the solution was added to the chitosan solution at a rate of 150 µL.min^-1^. To maintain the pH constant, 3.1 mL HCl 1 M was added simultaneously at a rate of 8 µL.min^-1^. The mixture was maintained at 30°C during the addition of the p-NCS-Bz-DFO and HCl solution and was stirred at 30°C during at least 2 hours after the end of the addition of the p-NCS-Bz-DFO solution.

**
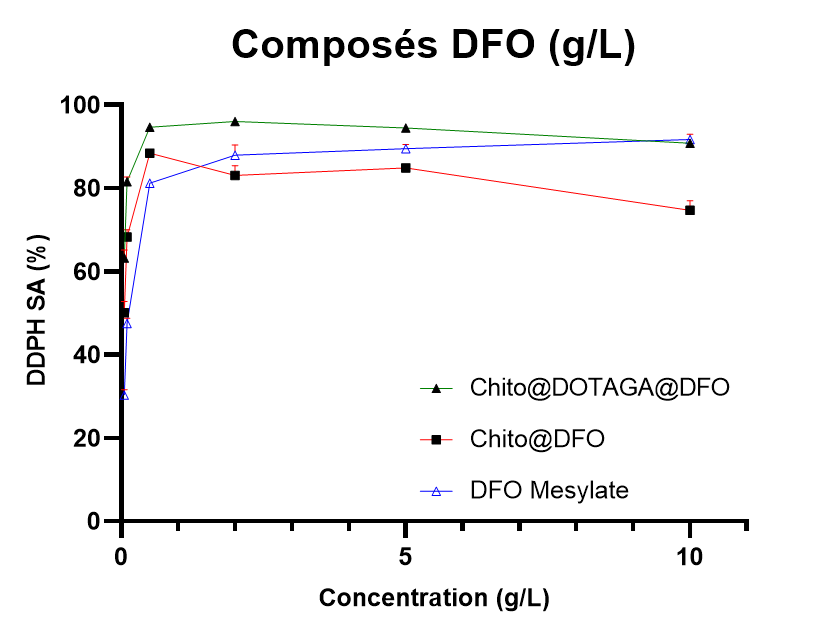
**

**Figure S7.** Antioxidant properties of DFO mesylate (empty triangle) and DFO-grafted chitosans (triangle: Chito@DOTAGA@DFO, square: Chito@DFO) measured by DDPH assay, after 1 h of reaction.

**
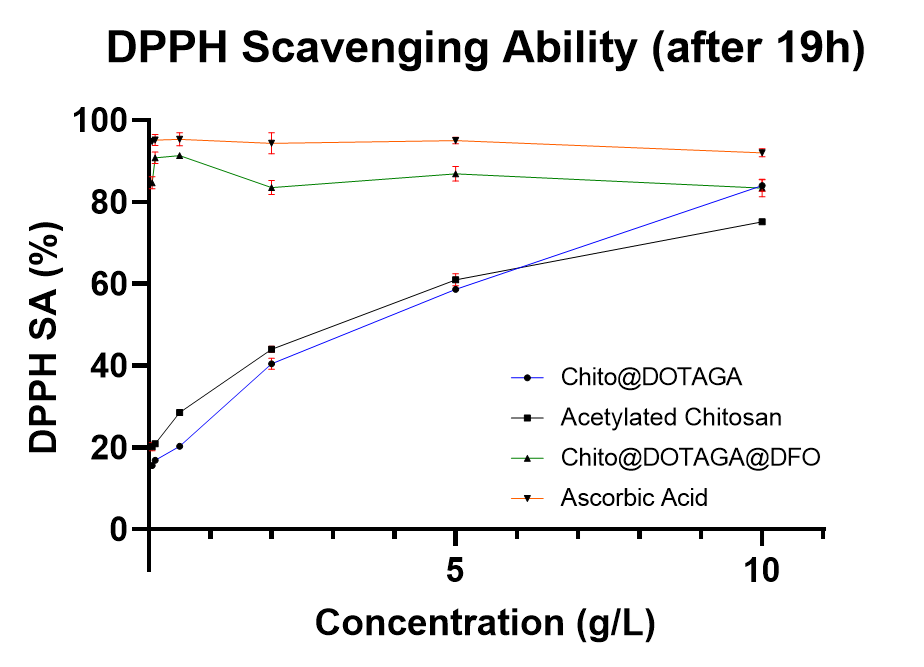
**

**Figure S8.** DPPH scavenging ability of functionalized chitosan (square: acetylated chitosan DA=29%; circle: Chito@DOTAGA; triangle: Chito@DOTAGA@DFO) and ascorbic acid (upside-down triangle) after 19h of reaction.

**Iron extraction efficiency versus Deferiprone.**

**Table S2.** Iron concentrations (^56^Fe) measured by ICP-MS in control samples without polymers (in triplicates)

|  | 1.8 µM Deferiprone | | | 14.4 µM Deferiprone | | |
| --- | --- | --- | --- | --- | --- | --- |
| Replicates | 1 | 2 | 3 | 1 | 2 | 3 |
| [Fe] in initial solution (ppb) | 27.95 | 27.74 | 25.66 | 27.41 | 28.44 | 27.96 |
| [Fe] in undernatant (ppb) | 27.81 | 28.90 | 30.36 | 29.80 | 30.26 | 29.58 |
| **% Recovery in undernatant** | **99.5%** | **104.2%** | **118.3%** | **108.7%** | **106.4%** | **105.8%** |

**Table S3.** Protonation and complexation constants of DOTA, Deferiprone and DFO in literature and recalculated at pH 7.4

|  | pKa | Ligand form  involved in complexation | Predominant form  at pH 7.4 | Log Kc | Log K_app7.7_ |
| --- | --- | --- | --- | --- | --- |
| DOTA | 4.13  4.60  9.72  11.9  *[29]* | L^4-^ | LH_2_^2-^ | (ML-): 29.4  *[26]* | (ML-):  7.8 |
| DFO | 8.71  9.21  9.94  10.97  *[25]* | L^3-^ | LH_4_^+^ | (MHL^+^): 41.8  *[25]* | (MHL^+^):  13.9 |
| Def. | 3.56  9.64  *[32]* | 3 L^-^ | LH | (ML_3_):  36.7  *[32]* | (ML_3_):  7.8 |

Given that complexation experiments were realized in physiological conditions (pH 7.4 ± 0.5), it is necessary to calculate complexation constants at this pH to compare the chelating agents. In literature, complexation constants are given for the species involved in the reaction (L^4-^ for DOTA and L^3-^ for DFO). However, at pH 7.4, DOTA is mainly on its form LH_2_^2-^ and DFO on its form LH^4+^. Complexation equation at pH 7.4 were written with the predominant ligand species at this pH and protonation constants were taken into account to calculate the apparent complexation constant at this pH.

***Ex vivo* efficacy of iron extraction by hemodialysis.**

**Table S4.** Stability of dialysate solution with or without Chito@DOTAGA@DFO over a 4-hour dialysis session

|  | **Hemosol B0** | | **Hemosol B0 + Chito@DOTAGA@DFO** | |
| --- | --- | --- | --- | --- |
| **Time of dialysis** | T0 | 4h | T0 | 4h |
| **pH** | 7.6 | 7.9 | 7.6 | 8.0 |
| **Osmolarity (mOsm/L)** | 258 | 255 | 246 | 251 |
| **Viscosity** | 1.3 mPa.s  at 19.2°C | 1.1 mPa.s  at 24.8°C | 1.4 mPa.s  at 19.8°C | 1.3 mPa.s  at 24.4°C |
| **Visual aspect** | Clear and homogeneous solution | Clear and homogeneous solution | Slightly yellow, limpid and homogeneous solution | Slightly yellow, limpid and homogeneous solution |

**Table S5.** Summary of metal concentration in initial solution (IS), undernatant (UN) and supernatant (SN) in the competition experiment with deferiprone, analyzed by ICP-MS (raw data)

| **Sample Id** | **Fe 56 Helium KED (ppb)** | | |
| --- | --- | --- | --- |
|  | **Replicate 1** | **Replicate 2** | **Replicate 3** |
| [IS1_Chito@DOTAGA](mailto:IS1_Chito@DOTAGA) | 5.388 | 0.017 | 0.006 |
| IS2_Chito@DOTAGA@DFO | -0.051 | -0.021 | -0.039 |
| IS3_1.8 µM Def | 27.948 | 27.745 | 25.659 |
| IS4_14.4 µM Def | 27.414 | 28.437 | 27.962 |
| IS5_Chito@DOTAGA_1.8 µM Def | 25.862 | 26.210 | 31.915 |
| [IS6_Chito@DOTAGA_14.4 µM Def](mailto:IS6_Chito@DOTAGA_14.4%20µM%20Def) | 27.163 | 27.648 | 26.935 |
| [IS7_Chito@DOTAGA@DFO_1.8 µM Def](mailto:IS5_Chito@DOTAGA@DFO_1.8%20µM%20Def) | 28.071 | 29.729 | 26.416 |
| [IS8_Chito@DOTAGA@DFO_14.4 µM Def](mailto:IS8_Chito@DOTAGA@DFO_14.4%20µM%20Def) | 38.340 | 29.028 | 29.104 |
| UN1_Chito@DOTAGA | 2.012 | 2.064 | 0.482 |
| UN2_Chito@DOTAGA@DFO | 0.559 | 1.270 | 0.905 |
| UN3_1.8 µM Def | 27.808 | 28.903 | 30.356 |
| UN4_14.4 µM Def | 29.798 | 30.262 | 29.582 |
| UN5_Chito@DOTAGA_1.8 µM Def | 27.257 | 28.841 | 27.405 |
| UN6_Chito@DOTAGA_14.4 µM Def | 28.919 | 29.542 | 28.991 |
| UN7_Chito@DOTAGA@DFO_1.8 µM Def | 2.129 | 1.756 | 6.397 |
| UN8_Chito@DOTAGA@DFO_14.4 µM Def | 3.093 | 2.211 | 4.693 |
| [SN1_Chito@DOTAGA](mailto:IS1_Chito@DOTAGA) | 3.216 | 3.300 | 3.189 |
| SN2_Chito@DOTAGA@DFO | 3.655 | 3.572 | 5.229 |
| SN3_1.8 µM Def | 13.391 | 36.533 | 32.634 |
| SN4_14.4 µM Def | 10.756 | 24.672 | 34.738 |
| SN5_Chito@DOTAGA_1.8 µM Def | 21.969 | 25.252 | 20.245 |
| [SN6_Chito@DOTAGA_14.4 µM Def](mailto:IS6_Chito@DOTAGA_14.4%20µM%20Def) | 21.682 | 16.744 | 17.243 |
| [SN7_Chito@DOTAGA@DFO_1.8 µM Def](mailto:IS5_Chito@DOTAGA@DFO_1.8%20µM%20Def) | 51.454 | 43.448 | 51.155 |
| [SN8_Chito@DOTAGA@DFO_14.4 µM Def](mailto:IS8_Chito@DOTAGA@DFO_14.4%20µM%20Def) | 40.623 | 39.290 | 41.708 |

**Table S6.** Summary of metal concentration in dialysate and effluent bag during hemodialysis, analyzed by ICP-MS (raw data)

|  | **Sample Id** | **Cu 63 Helium KED (ppb)** | **Cu 65 Helium KED (ppb)** | **Fe 56 Helium KED (ppb)** | **Fe 54 Helium KED (ppb)** |
| --- | --- | --- | --- | --- | --- |
| Dialysate | SepteX_Chito@DOTAGA@DFO_T0-1 Dialysate | 1.94 | 0.83 | 14.47 | 12.65 |
|  | SepteX_Chito@DOTAGA@DFO_T0-2 Dialysate | 3.31 | 3.60 | 13.05 | 10.21 |
| Effluent bag | SepteX_Chito@DOTAGA@DFO_T1h-1 Effluent | 245.97 | 246.27 | 889.36 | 891.66 |
|  | SepteX_Chito@DOTAGA@DFO_T1h-2 Effluent | 253.87 | 252.07 | 930.29 | 934.89 |
|  | SepteX_Chito@DOTAGA@DFO_T2h-1 Effluent | 254.81 | 253.66 | 925.16 | 920.92 |
|  | SepteX_Chito@DOTAGA@DFO_T2h-2 Effluent | 257.76 | 254.21 | 936.27 | 936.71 |
|  | SepteX_Chito@DOTAGA@DFO_T3h-1 Effluent | 252.18 | 251.01 | 938.28 | 961.35 |
|  | SepteX_Chito@DOTAGA@DFO_T3h-2 Effluent | 244.45 | 244.16 | 912.67 | 912.99 |
|  | SepteX_Chito@DOTAGA@DFO_T4h-1 Effluent | 165.79 | 164.35 | 652.53 | 647.51 |
|  | SepteX_Chito@DOTAGA@DFO_T4h-2 Effluent | 170.31 | 167.68 | 671.15 | 675.02 |
| Dialysate | SepteX_Control_T0-1 Dialysate | 0.86 | 0.91 | 5.95 | 3.50 |
|  | SepteX_Control_T0-2 Dialysate | 1.26 | 1.31 | 7.67 | 4.46 |
| Effluent bag | SepteX_Control_T1h-1 Effluent | 223.27 | 223.47 | 842.15 | 839.53 |
|  | SepteX_Control_T1h-2 Effluent | 215.56 | 216.34 | 802.23 | 812.30 |
|  | SepteX_Control_T2h-1 Effluent | 215.56 | 215.37 | 820.83 | 842.10 |
|  | SepteX_Control_T2h-2 Effluent | 218.71 | 218.02 | 827.34 | 835.85 |
|  | SepteX_Control_T3h-1 Effluent | 202.50 | 204.93 | 763.59 | 759.99 |
|  | SepteX_Control_T3h-2 Effluent | 206.44 | 206.49 | 781.96 | 762.40 |
|  | SepteX_Control_T4h-1 Effluent | 138.13 | 138.47 | 528.97 | 527.50 |
|  | SepteX_Control_T4h-2 Effluent | 148.09 | 148.08 | 565.28 | 567.84 |
| Dialysate | HF1400_Chito@DOTAGA@DFO_T0-1 Dialysate | 1.17 | 1.25 | 15.51 | 11.56 |
|  | HF1400_Chito@DOTAGA@DFO_T0-2 Dialysate | 1.56 | 1.64 | 23.76 | 20.81 |
| Effluent bag | HF1400_Chito@DOTAGA@DFO_T1h-1 Effluent | 9.12 | 9.11 | 31.35 | 32.38 |
|  | HF1400_Chito@DOTAGA@DFO_T1h-2 Effluent | 8.92 | 9.02 | 34.46 | 33.17 |
|  | HF1400_Chito@DOTAGA@DFO_T2h-1 Effluent | 8.93 | 9.37 | 35.09 | 34.52 |
|  | HF1400_Chito@DOTAGA@DFO_T2h-2 Effluent | 8.77 | 8.99 | 30.73 | 31.44 |
|  | HF1400_Chito@DOTAGA@DFO_T3h-1 Effluent | 8.67 | 8.64 | 38.03 | 38.54 |
|  | HF1400_Chito@DOTAGA@DFO_T3h-2 Effluent | 8.60 | 8.78 | 32.36 | 32.09 |
|  | HF1400_Chito@DOTAGA@DFO_T4h-1 Effluent | 7.34 | 7.77 | 34.23 | 29.73 |
|  | HF1400_Chito@DOTAGA@DFO_T4h-2 Effluent | 7.39 | 7.31 | 34.87 | 33.66 |
| Dialysate | HF1400_Control_T0-1 Dialysate | 0.03 | 0.03 | 5.47 | 5.21 |
|  | HF1400_Control_T0-2 Dialysate | 0.03 | 0.03 | 11.71 | 10.49 |
| Effluent bag | HF1400_Control_T1h-1 Effluent | 6.08 | 6.49 | 27.77 | 24.48 |
|  | HF1400_Control_T1h-2 Effluent | 3.68 | 4.06 | 23.67 | 23.27 |
|  | HF1400_Control_T2h-1 Effluent | 5.33 | 5.43 | 19.49 | 20.45 |
|  | HF1400_Control_T2h-2 Effluent | 3.26 | 3.72 | 19.84 | 17.69 |
|  | HF1400_Control_T3h-1 Effluent | 3.61 | 3.62 | 19.59 | 17.88 |
|  | HF1400_Control_T3h-2 Effluent | 3.39 | 3.52 | 20.85 | 19.93 |
|  | HF1400_Control_T4h-1 Effluent | 2.31 | 2.38 | 15.39 | 13.37 |
|  | HF1400_Control_T4h-2 Effluent | 2.97 | 3.28 | 17.00 | 14.98 |

**Table S7.** Summary of metal concentration (ppb) and metal content (µg) after correction in dialysate (t0) and effluent bag (1 h to 4 h) during hemodialysis, analyzed by ICP-MS.

|  | **Time (h)** | **Volume of dialysate (L)** | **Raw metallic concentration (ppb)** | | | | **Corrected metallic concentration (ppb)** | | | | **Corrected metallic content (µg)** | | | |
| --- | --- | --- | --- | --- | --- | --- | --- | --- | --- | --- | --- | --- | --- | --- |
|  |  |  | **Cu mean (ppb)** | **RSD Cu (ppb)** | **Fe mean (ppb)** | **RSD Fe (ppb)** | **Cu corrected (ppb)** | **RSD Cu corrected (pbb)** | **Fe corrected (ppb)** | **RSD Fe corrected (ppb)** | **Cu corrected (µg)** | **RSD Cu corrected (µg)** | **Fe corrected (µg)** | **RSD Fe corrected (µg)** |
| SEPTEX-Chito@DOTAGA@DFO | 0 | 0.0 | 2.42 | 1.28 | 12.60 | 1.77 | NA | NA | NA | NA | NA | NA | NA | NA |
|  | 1 | 0.5 | 249.54 | 4.02 | 911.55 | 24.38 | 247.12 | 1.15 | 898.95 | 2.56 | 123.56 | 0.58 | 449.48 | 1.28 |
|  | 2 | 1.0 | 255.11 | 1.83 | 929.76 | 7.96 | 252.69 | 0.88 | 917.17 | 1.56 | 252.69 | 0.88 | 917.17 | 1.56 |
|  | 3 | 1.5 | 247.95 | 4.23 | 931.32 | 23.34 | 245.53 | 1.17 | 918.72 | 2.51 | 368.29 | 1.76 | 1378.09 | 3.76 |
|  | 4 | 2.0 | 167.03 | 2.58 | 661.55 | 13.57 | 164.61 | 0.98 | 648.96 | 1.96 | 329.22 | 1.96 | 1297.91 | 3.92 |
| SEPTEX-Control | 0 | 0.0 | 1.08 | 0.23 | 5.39 | 1.82 | NA | NA | NA | NA | NA | NA | NA | NA |
|  | 1 | 0.5 | 219.66 | 4.29 | 824.05 | 19.84 | 218.58 | 1.06 | 818.66 | 2.33 | 109.29 | 0.53 | 409.33 | 1.16 |
|  | 2 | 1.0 | 216.92 | 1.70 | 831.53 | 9.35 | 215.83 | 0.70 | 826.14 | 1.67 | 215.83 | 0.70 | 826.14 | 1.67 |
|  | 3 | 1.5 | 205.09 | 1.87 | 766.98 | 10.10 | 204.01 | 0.73 | 761.59 | 1.73 | 306.01 | 1.09 | 1142.38 | 2.59 |
|  | 4 | 2.0 | 143.19 | 5.65 | 547.39 | 22.16 | 142.11 | 1.21 | 542.00 | 2.45 | 284.22 | 2.43 | 1084.00 | 4.90 |
| HF1400-Chito@DOTAGA@DFO | 0 | 0.0 | 1.41 | 0.23 | 17.91 | 5.44 | NA | NA | NA | NA | NA | NA | NA | NA |
|  | 1 | 0.5 | 9.04 | 0.10 | 32.84 | 1.31 | 7.64 | 0.29 | 14.93 | 1.30 | 3.82 | 0.14 | 7.46 | 0.65 |
|  | 2 | 1.0 | 9.01 | 0.25 | 32.95 | 2.18 | 7.61 | 0.35 | 15.04 | 1.38 | 7.61 | 0.35 | 15.04 | 1.38 |
|  | 3 | 1.5 | 8.67 | 0.08 | 35.26 | 3.51 | 7.27 | 0.28 | 17.35 | 1.50 | 10.90 | 0.41 | 26.02 | 2.24 |
|  | 4 | 2.0 | 7.45 | 0.21 | 33.12 | 2.32 | 6.04 | 0.33 | 15.21 | 1.39 | 12.09 | 0.67 | 30.43 | 2.78 |
| HF1400-Control | 0 | 0.0 | 0.03 | 0.00 | 8.22 | 3.37 | NA | NA | NA | NA | NA | NA | NA | NA |
|  | 1 | 0.5 | 5.08 | 1.41 | 24.80 | 2.05 | 5.05 | 0.59 | 16.58 | 1.16 | 2.52 | 0.30 | 8.29 | 0.58 |
|  | 2 | 1.0 | 4.44 | 1.11 | 19.37 | 1.19 | 4.41 | 0.53 | 11.15 | 1.07 | 4.41 | 0.53 | 11.15 | 1.07 |
|  | 3 | 1.5 | 3.53 | 0.10 | 19.56 | 1.24 | 3.50 | 0.16 | 11.34 | 1.07 | 5.25 | 0.25 | 17.02 | 1.61 |
|  | 4 | 2.0 | 2.73 | 0.47 | 15.19 | 1.49 | 2.70 | 0.34 | 6.97 | 1.10 | 5.40 | 0.69 | 13.94 | 2.20 |
